# Neuromorphic-computing-based adaptive learning using ion dynamics in flexible energy storage devices

**DOI:** 10.1093/nsr/nwac158

**Published:** 2022-08-13

**Authors:** Shufang Zhao, Wenhao Ran, Zheng Lou, Linlin Li, Swapnadeep Poddar, Lili Wang, Zhiyong Fan, Guozhen Shen

**Affiliations:** State Key Laboratory for Superlattices and Microstructures, Institute of Semiconductors, Chinese Academy of Sciences, and Center of Materials Science and Optoelectronic Engineering, University of Chinese Academy of Sciences, Beijing 100083, China; State Key Laboratory for Superlattices and Microstructures, Institute of Semiconductors, Chinese Academy of Sciences, and Center of Materials Science and Optoelectronic Engineering, University of Chinese Academy of Sciences, Beijing 100083, China; State Key Laboratory for Superlattices and Microstructures, Institute of Semiconductors, Chinese Academy of Sciences, and Center of Materials Science and Optoelectronic Engineering, University of Chinese Academy of Sciences, Beijing 100083, China; State Key Laboratory for Superlattices and Microstructures, Institute of Semiconductors, Chinese Academy of Sciences, and Center of Materials Science and Optoelectronic Engineering, University of Chinese Academy of Sciences, Beijing 100083, China; Department of Electronic and Computer Engineering, The Hong Kong University of Science and Technology, Hong Kong, China; State Key Laboratory for Superlattices and Microstructures, Institute of Semiconductors, Chinese Academy of Sciences, and Center of Materials Science and Optoelectronic Engineering, University of Chinese Academy of Sciences, Beijing 100083, China; Department of Electronic and Computer Engineering, The Hong Kong University of Science and Technology, Hong Kong, China; School of Integrated Circuits and Electronics, Beijing Institute of Technology, Beijing 100081, China

**Keywords:** neuromorphic computing, MXene, flexible device, high accuracy, adaptability, ion dynamics

## Abstract

High-accuracy neuromorphic devices with adaptive weight adjustment are crucial for high-performance computing. However, limited studies have been conducted on achieving selective and linear synaptic weight updates without changing electrical pulses. Herein, we propose high-accuracy and self-adaptive artificial synapses based on tunable and flexible MXene energy storage devices. These synapses can be adjusted adaptively depending on the stored weight value to mitigate time and energy loss resulting from recalculation. The resistance can be used to effectively regulate the accumulation and dissipation of ions in single devices, without changing the external pulse stimulation or preprogramming, to ensure selective and linear synaptic weight updates. The feasibility of the proposed neural network based on the synapses of flexible energy devices was investigated through training and machine learning. The results indicated that the device achieved a recognition accuracy of ∼95% for various neural network calculation tasks such as numeric classification.

## Teaser

A novel neuromorphic device based on a flexible MXene energy storage device was designed that can achieve a self-adaption neural network to avoid the loss of time and energy caused by recalculation.

## INTRODUCTION

Neuromorphic computing is an information-processing model that emulates the efficiency, versatility and flexibility of the human brain [1,2]. Artificial synaptic devices have been used to construct artificial neural networks (ANNs) for neuromorphic calculations [3–5]. However, the training and learning of neural computing determines the adjustment direction of the weights based on the gradient of the error function. And the weights are subsequently adjusted using an artificial synaptic device. Moreover, using the known data for training and adapting to the most unmarked real-time changing input data is challenging due to fixed weights [6]. Therefore, continuously adapting neural networks to environmental changes in applications remains challenging. If an artificial neuromorphic chip is appropriate for real-time applications with a massive amount of unlabeled data, the neural plasticity of the brain, especially its synaptic plasticity, should be simulated in electronic devices [7]. Various resistive and phase-change memories have been used to simulate synapses [8–10]. However, in these memory techniques, synaptic intensity is reconstructed primarily through software programming and varying the pulse time, which may result in low efficiency and high energy consumption in neuromorphic calculations [11]. Furthermore, the following challenges occur in conventional memory: excess write noise, write non-linearity (NL) and diffusion under zero bias pressure.

Similar to synapses, charge-based energy storage devices can perform conductance modulation and retention under low-energy conditions [12]. The unique characteristics of ion shuttles inspired novel artificial synaptic simulation of synaptic gap information transmission for energy storage devices. Experimental results have revealed that a battery-like switch device can be used as an artificial synapse for brain-power-level low-energy calculation [13,14]. However, achieving selective and linear weight updates to satisfy the requirements for neuromorphic computing with high-accuracy and self-adaption remains challenging.

In this study, a novel hardware neural network based on a tunable flexible MXene energy storage (FMES) system is proposed. The synaptic weights, represented by Δ*w* in machine learning, could be changed without varying the external stimulus. Coupling MXene and gel electrolyte could control the accumulation and dissipation of ions by tuning the resistance, which modulates Δw. Results of machine-learning simulations prove that the synapse based on tunable FMES device can be used in neuromorphic calculation tasks (e.g. number classification and pattern recognition). For a dataset of handwritten patterns, by changing the resistance and seeking the best learning rate, the proposed system achieved a recognition accuracy of ∼95%. Furthermore, the FMES device can be adaptively adjusted according to the stored weight value. Therefore, the time and energy loss caused by recalculation was prevented. This study is crucial for simulating human memory features and artificial neural systems.

## RESULTS

### Tunable FMES device

**Figure 1.**
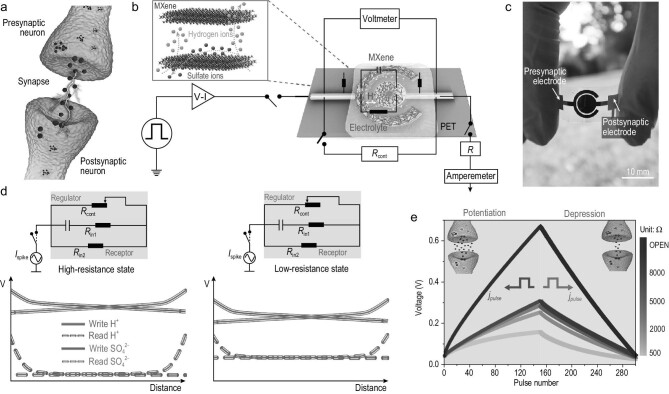
Structure and electronic states of an FMES device. (a) Structural and functional illustration of the biological synapse. (b) Illustration of the FMES device. Inset shows dynamic diffusion process. (c) Optical image of the FMES device. (d) Schematic explaining the corresponding circuit diagram and the decoupling of the read and write operations. (e) Monitored postsynaptic current under several sequential voltage pulses.

Supercapacitors exhibit considerable potential as energy devices for the simulation of synaptic behaviors based on the energy storage and voltage change caused by ionic movements and adsorption [13,15]. As displayed in Fig. 1a, an FMES device was integrated into a resistance-controlled system to construct a synaptic device. The system comprises a flexible postsynaptic electrode and an MXene nanoflake and is bounded by an MXene flexible presynaptic electrode through an electrolyte (Fig. 1b). This FMES device exhibits considerable potential for manufacturing low-cost flexible artificial synaptic systems (Fig. 1c) to integrate neuromorphic learning and computing in neural electrode arrays, implantable prosthetics or any other large-area flexible electronic system (Supplementary Fig. 1). Transmission electron microscopy images of the few-layered MXene nanoflakes (Supplementary Fig. 2) [16] exhibited excellent lattice fringes (Supplementary Fig. 3), indicating high crystallinity. Such high conductivity and low ion diffusion barriers in Ti_3_C_2_T_x_ MXene contribute to its surface adsorption (Supplementary Fig. 4). When a set of excitatory current spikes (*I_spike_*) is applied to the FMES system, cations in the electrolyte move toward the electrodes and are adsorbed on their surfaces. Therefore, the amount of charge that can be stored increases. When excitatory current *I_spike_* is removed, because of the long self-discharge time, the output voltage of the FMES device can be maintained in this state for a long time after a short decrease. When this current is applied to the device again, ions are continuously attracted to the electrodes to charge the device. This phenomenon results in a higher output voltage than in previous processes. In this process, the voltage of the FMES system represents the synaptic weight of the connection between two neurons, which is a basic feature of an artificial synapse (Supplementary Fig. 5). Furthermore, the I–V curves of the FMES device vary with the sweep rates (Supplementary Fig. 6). This phenomenon confirmed that the FMES system can be used as a synaptic device, and its storage state can be modulated by the amplitude, duration and frequency of the input pulse (Supplementary Figs 7 and 8 and Supplementary Note 1).

Although FMES devices exhibit non-volatile memory functions, an FMES device was developed by introducing a resistance to adapt to the frequent weight updates of neuromorphic memory calculations. Without changing the external pulse stimuli, non-volatile weight-change characteristics of biological synapses are realized by regulating the movement and accumulation of cations in the electrolyte (Fig. 1d and Supplementary Note 2). Figure 1e displays the potentiation and depression behaviors of the artificial synapses. These synapses were distributed in various conductance regions with excellent linearity based on various resistance rates. These results revealed that the synaptic weight could be effectively adjusted by changing the resistance, to realize neuromorphic memory calculations, which reflects the building blocks of neuromorphic computing.

### Principles and characteristics of the FMES device

**Figure 2.**
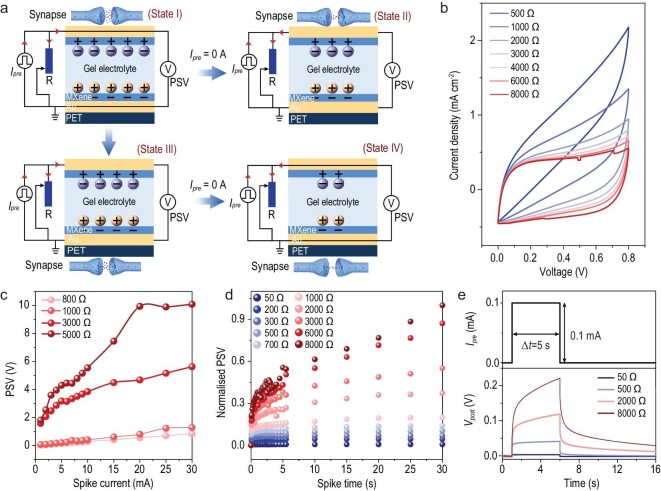
Principles and characteristics of the FMES device. (a) Operating principle circuit diagram of the FMES device. (b) I–V curves measured in sweep cycles of 0 to 0.8 V with 50 mV s^–1^ sweep rate. (c and d) Change in the PSV as a function of (c) presynaptic pulse intensity and (d) width, with various resistance levels. (e) Dynamic PSV change curves of the FMES device where the resistance increases at the same spike time.

Figure 2a displays the circuit structure of the entire FMES system and the resulting ionic motion. This design facilitates the FMES device with a proper parallel resistance and enables a change in its synaptic properties [17]. When a current *I*_spike_ (*I* = *I_1_*_ _+ *I_2_*) is applied to the presynaptic terminal, currents *I_1_* and *I_2_* flow through the regulating resistor and the FMES in the synaptic device (state I). Under current spike (I_2_), ions in the FMES device move to positive and negative poles and are adsorbed on the surface of MXene to realize charge storage (Q = $\int_{0}^{t}{{{I}_2dt}}$). At this moment, a postsynaptic voltage (PSV) U = Q/C appears at the postsynaptic terminal. When the resistance value decreased, Q stored in the FMES device decreased; consequently, the output PSV of the entire system also decreases. Therefore, a change in the resistance can change the synaptic behavior of the entire system during presynaptic current (*I_pre_*) stimulation (state III). When *I_spike_* is removed, because of self-discharging, the two carriers adsorbed on the surface of MXene are re-released into the electrolyte. This phenomenon results in an output current flowing to the regulating resistor and consuming the charge stored in the FMES device [13,14,18]. The process results in a PSV reduction of the entire system (state II). Because the PSV is related to the residual charge in the FMES system, according to the preceding assumption, the following expression can be deduced:

$$\begin{equation*}
{{u}}({{t}}) = \frac{1}{C} \cdot \left( {{Q}_0 - \int_{0}^{t}{{\frac{{u(t)}}{R}dt}}} \right),
\end{equation*}$$

where *u(t)* is the PSV, *t* is the forgetting time of the synaptic system after *I*_spike_ is removed, *Q_0_* is the amount of charge stored in the FMES system when *I_spike_* is removed, and *R* is the value of the regulating resistance. Because of the constant *t*, the PSV of the synaptic system decreases with a decrease in *R*. When the value of the regulating resistance decreases, the PSV of the artificial synaptic system also decreases, which increases the forgetting speed (state IV). Thus, the synaptic behavior of the entire artificial synaptic system can be controlled by adjusting the regulating resistance.

When the resistance is tuned from 500 to 8000 Ω, the magnitude of the synaptic current decreases from 1.6 to 0.47 mA at 0.6 V (Fig. 2b). Furthermore, the PSV exhibits a considerable increase from 0.12 to 0.74 V at 0.5 mA, which is a typical trait of synaptic plasticity [19]. To detail the changes in the synaptic plasticity in the FMES system and the effects of the changes resulting from regulating the resistance on the synaptic learning behavior, excitatory presynaptic currents with the same stimulating time (1 ms) and various intensities were applied to investigate the changes in the PSV that correspond to various resistance states (Fig. 2c). The results indicate that the PSV increases with an enhancement in the presynaptic current intensity. In addition to the effect of the stimulation intensity on the PSV of the synaptic system, the PSV increases with an increase in resistance (Supplementary Fig. 9) [20]. Moreover, the PSV value increases with an enhancement in the pulse width (Fig. 2d). When the pulse width was fixed, the PSV increased with an increase in resistance, and vice versa (Fig. 2e). These results indicate that the resistance can regulate not only synaptic plasticity but also the activity of the synaptic system. Generally, endurance determines the long-term working capability of artificial synaptic devices. To evaluate the durability of the device (Supplementary Fig. 10 and Supplementary Note 3) and the stability between devices (Supplementary Fig. 11 and Supplementary Note 3), we fabricated an array, displayed as in Supplementary Fig. 11a. Durability tests on the device and the difference of the device array revealed that the variability of devices is limited (Supplementary Fig. 11b–d).

### Weight updates

**Figure 3.**
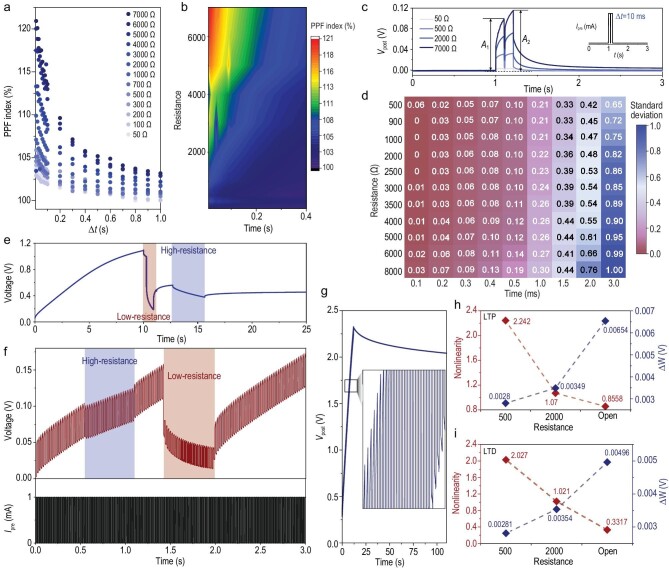
Weight updates to various resistance conditions. (a) Plot and (b) heat map of the PPF index (A_2_/A_1_) and interval time Δt, where A_1_ and A_2_ are the first and second EPSV. (c) PSV curves triggered by two consecutive excitatory current pulses in FMES device. (d) The standard deviation of the autocorrelation function of the corresponding PSV curve. (e) Dynamic LTP characteristic curves for the FMES device under a prolonged condition of presynaptic current stimulation. (f) Dynamic learning behavior of FMES device. (g) The EPSV response under continuous excitatory current stimulations (500 Hz, 1 mA). Calculated non-linearity and Δw of (h) LTP and (i) LTD as the function of resistance.

In the human brain, learning and forgetting performance depends on the management of synaptic weights [21] (e.g. synaptic plasticity). This management can be categorized as either short-term or long-term synaptic plasticity (STSP or LTSP) [22]. STSP is a key factor in neuromorphic computing, and paired-pulse facilitation (PPF) is a typical STSP feature of a synapse [23]. Figure 3a and b displays the PPF under various resistances and time intervals (Δ*t*). The results indicate that the excitatory postsynaptic voltage (EPSV) caused by the second spike increased when it followed the previous spike. The peak voltage of the EPSV activated by the second contact–separation spike (A_2_ = 0.115 V, 7000 Ω) was higher than that activated by the first spike (A_1_ = 0.094 V, 7000 Ω) at Δ*t* = 0.01 s (Fig. 3c). In addition to the influence of the time interval, the PPF (*A*_2_/*A*_1_) index decayed rapidly with the decrease in resistance, and the time of decay to 100% accelerated [24]. This phenomenon directly indicates that increasing the resistance value can also increase the potential plasticity and simultaneously improve memorizing capability as well as reduce the forgetting speed of the FMES system. According to the calculation of the autocorrelation function of the corresponding PSV curve under various resistance states presented in Fig. 2d, the standard deviation of the curve was extracted to prove the effect of the change in resistance on the memory ability of the FMES system (Supplementary Fig. 12 and Supplementary Note 4). With continuous improvement of resistance and stimulation time, the memorizing capability of the FMES system improved gradually (Fig. 3d). Figure 3e and Supplementary Fig. 13 indicate that the memory time is particularly long, and the system exhibits an excellent LTSP. As displayed in Fig. 3f, the learning behavior of the FMES system was studied by adjusting the change in the regulating resistance under the stimulation of a presynaptic stimulation current (1 mA) with a fixed frequency of 500 Hz. Moreover, the learning speed could be changed by adjusting the resistance. As the resistance increased, learning became easier. When the resistance was in the open state, the PSV of the FMES system increased rapidly again, which proved the associative memory of the artificial synapse.

To prove the LTSP behavior of the FMES system, a continuous presynaptic current (500 Hz, 1 mA) was used to stimulate the system without considering the change in resistance. Long-term forgetting (>100 s) was subsequently performed. Notably, the PSV remained almost unchanged during the forgetting process (Fig. 3g). Furthermore, during the learning process, the learning efficiency improved by adjusting the synaptic weight. LTSP is a key component of memory control in hippocampal neurons and is primarily composed of the long-term potentiation and depression (LTP and LTD) of the synaptic weights [25]. In contrast to reported devices, the LTP/LTD performance of the proposed FMES device was optimized by adjusting the resistance state to regulate the ion transmission and accumulation behavior. This modulation of the weight updates, which corresponds to various resistance states, was tested experimentally and used to define synaptic plasticity for the simulation of ANNs. Figure 1e and Supplementary Fig. 14 display the LTP/LTD characteristics of the FMES devices measured by the application of 150 potentiation pulses (1 mA) and 150 depression pulses (−1 mA) under various resistance states, respectively. The LTP/LTD performance strongly depended on the resistance state; that is, during the learning process, the slope of the PSV growth changed with a change in regulatory resistance. The dynamic range (*W*_max_/*W*_min_) values of the FMES device under the resistances of 500, 1000 and 8000 Ω and the open state were >6, which is the minimum value required for a successful completion of pattern recognition tasks (Supplementary Fig. 15).

The NL values at various resistance values under LTP and LTD were calculated (Fig. 3h and i; the fitting method is detailed in Supplementary Note 5), and the influence of the NL on the learning efficiency of the FMES device-based neural network was analyzed. At various resistance values, the device exhibited excellent synaptic behavior in the LTP and LTD regions (i.e. the highest effective number of states (*N*_Seff_) and low NL values) [26]. The resistance state affected the weight change. High resistance values induced a higher weight change than lower resistance values, which decreased the NL in the LTP and LTD regions. This phenomenon indicated that the learning rate gradually increased. Furthermore, degradation was not observed on the proposed device during the bending tests, which indicated a reliable LTP/LTD behavior (Supplementary Fig. 16). This phenomenon enables the facile integration of neuromorphic computation and learning into neural electrode arrays, implantable prosthetics and any other flexible large-area electronic system [13,27].

### Neuromorphic pattern-recognition accuracy

**Figure 4.**
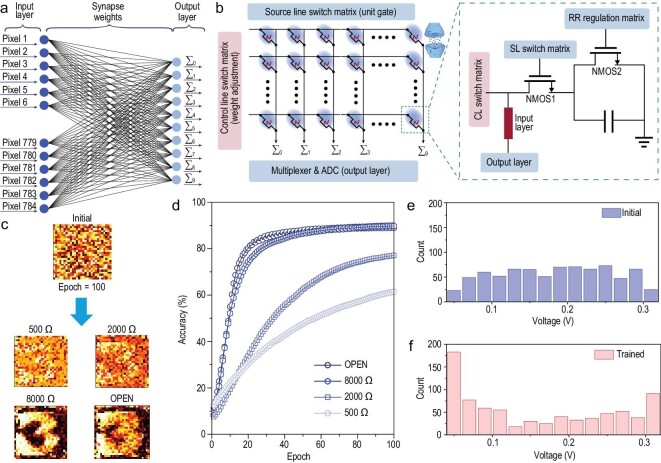
Accuracy of neuromorphic computation. (a) Tri-layer neural network structure. (b) Hardware neural network comprising FMES devices. (c) Mapping a representative input digit of 784 synaptic weights connected to the output digit ‘3’ at initial and various resistance states of training. (d) Comparison of the classification accuracy rates at initial and 100 training cycles. Distribution of the FMES devices’ PSV (e) before and (f) after training.

A single-layer perceptron model was designed to classify handwritten digits (adopted from the Modified National Institute of Standards and Technology (MNIST) dataset) in 28 × 28 pixel images, and the back-propagation algorithm was used for supervised learning (see Supplementary Note 5) [28]. Figure 4a displays the neural network for learning the MNIST data, which were categorized into three layers, namely 784, 100 and 10 neurons in the input neural, hidden and output layers, respectively. Supplementary Fig. 17 displays the specific operation mode of the simulation process (Supplementary Note 5) [22,29]. Figure 4b displays an example of the 2T1C cell structure used for circuit implementation, in which NMOS2 regulates the resistance of the synaptic system to control the electric currents for FMES device manipulation, which determines the amount of the weight change (learning rate; the verification results are displayed in Supplementary Fig. 18 and Supplementary Note 6) [30]. Supplementary Fig. 19 shows the feasibility of conceptual neural networks for MNIST digit patterns, image processing and recognition tasks.

Figure 4c displays the representative digit ‘3’ in the weight map of the ANN after 100 training epochs under four states of resistance. The other weight maps of the ANN are displayed in Supplementary Figs 20–22. At 8000 Ω resistance, the image of the weight mapping obtained by the weight updating formula was the clearest. As illustrated in Fig. 4d, the recognition accuracies for digital images of handwritten numbers at various resistance states, which reflect the synaptic plasticity in the FMES devices, were calculated. Notably, the proposed neural network (simulated by LTP/LTD pulses with a resistance value of 8000 Ω) achieved a maximum recognition accuracy of 90%, which was higher than that achieved in the open state (89%). For deployment in neural networks, improving the accuracy of the FMES device by using an algorithm is difficult because the learning rate achieved under resistance in the open state is faster than that at 8000 Ω. Thus, the optimal weight value could be missed during the learning process, resulting in a failure to achieve the best recognition accuracy.

Furthermore, the recognition rates for image processing and recognition are displayed in Supplementary Fig. 23. Figure 4e and f displays the distribution of synaptic weights in the neural network before and after learning, respectively. In the early stage of learning, the synaptic weights were random, and their distribution was average. After a period of learning and training, the synaptic weights in the network changed considerably, and the distribution began to gather on both sides regularly (Fig. 4f), which supports the applicability of the reported synaptic device to complex neural networks.

### Adaptive handwritten digit recognition simulation

**Figure 5.**
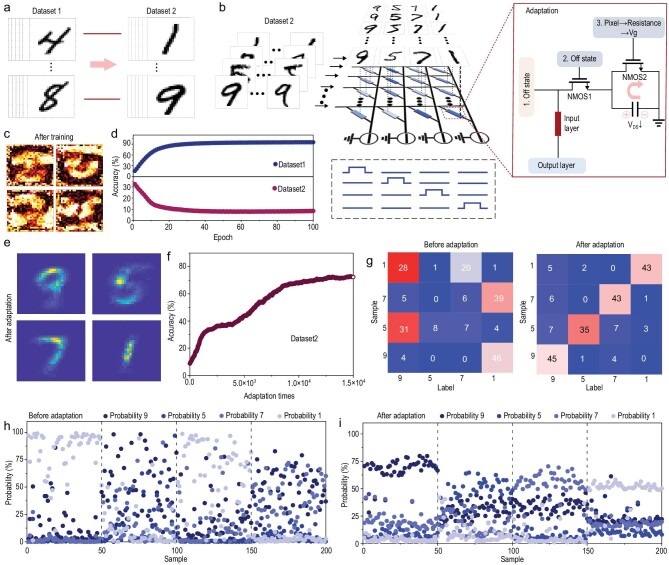
Adaptive handwritten digit recognition simulation. (a) Dataset1 and dataset2 extracted from MNIST. (b) Weight-mapping images of the ANN. (c) The recognition accuracy of dataset1 (top) and dataset2 (bottom). (d) Illustration of the adaptive adjustment of ANN weights. (e) After adaptation, the weight-mapping images of the ANN. (f) Relationship between the recognition accuracy of dataset2 by the ANN and the number of mapped images. (g) Classification and recognition of 200 samples by the ANN before and after adaptation. The probability of each sample in 200 samples belonging to four possible outcomes, (h) before and (i) after adaption.

The FMES device can be adjusted using an adaptive method based on the stored weight value, which avoids the time and energy loss caused by recalculation [31] (see Supplementary Note 5). To prove that the FMES system can self-adjust the weights in an adaptive method and then complete the switch of classification and identification similarity dataset types, we divided the MNIST dataset into two parts (Fig. 5a). The constructed neural network and detailed information are displayed in Supplementary Fig. 24. A schematic of the constructed ANN is displayed in Fig. 5b (similar to Fig. 4d). The weight-mapping images in the ANN after 100 training phases exhibited distinct digits ‘8’, ‘6’, ‘2’ and ‘4’. The ANN was able to successfully classify dataset1 (Fig. 5c). The corresponding accuracy is displayed in Fig. 5d (top), and can reach 95%. During training, we set the regulatory resistance of the FMES devices in the ANN to the open state, for two reasons: (i) according to Fig. 1e, the FMES system exhibits the widest range of the weight change in the open state. Therefore, the weight can be adjusted to the largest range; (ii) in the open state, the ANN exhibits high recognition accuracy for dataset1. Subsequently, we used the trained ANN to classify and recognize dataset2. The extremely low accuracy indicates that the trained ANN cannot be used to identify and classify dataset2 (Fig. 5d, bottom).

We extracted some samples from dataset2, converted the pixels into the regulatory resistance value, and realized the adaptive adjustment of the weight stored by the FMES device through continuous mapping images (Fig. 5b). The specific adaptive process and weight adjustment are detailed in Supplementary Note 7. Figure 5e displays the weight-mapping images of the post-adaptive ANN. The weight-mapping images in the ANN change from distinct digits ‘8’, ‘6’, ‘2’ and ‘4’ to distinct digits ‘9’, ‘5’, ‘7’ and ‘1.’ This indicates that after adaptation, the ANN can adapt to the recognition and classification of dataset2. Figure 5f reveals that as the number of images in dataset2 mapped to the trained ANN increases, the classification and recognition accuracy of dataset2 by the ANN continues to improve, with an enhancement from 8% to 73%. We used the adaptive ANN to identify the aforementioned 200 samples with results shown in Fig. 5g. Unlike the pre-adaptive ANN, the post-adaptive neural network results in superior sample recognition and higher accuracy [32]. The post-adaptive ANN predicts that the correct probability of the sample is improved, and the probability distribution is highly regular (Fig. 5h and i). Furthermore, we considered the performance of the device in the calculation to explain the conditions and results of the device in the neural morphology calculation, to a certain extent (Supplementary Fig. 25 and Supplementary Note 8). These results revealed that the automatic adjustment of the weight values of the trained ANN, through the adaptive method, can realize the classification and recognition of another dataset without retraining.

## CONCLUSION

In this study, FMES with resistance-tunable ion diffusion dynamics was developed to realize high-accuracy and self-adaptive neuromorphic computation. The accumulation of mobile ions in the electrolyte on the surface of MXene could be modulated by tuning the externally introduced resistance. Therefore, the value of Δ*w*, which can increase or decrease the learning rate for neuromorphic computing, can be effectively modulated. FMES device exhibited diverse synaptic characteristics through resistance-induced ion movement. In particular, the FMES device exhibited excellent linearity and symmetries under various resistance states, which assisted in frequent updating of the weights in neuromorphic calculations. Based on the resistance-tunable electronic characteristics, a high recognition rate of 95% was achieved for MNIST digit patterns. Moreover, the automatic adjustment of the weight value of the trained ANN, through the adaptive method, could realize the classification and recognition of another dataset without retraining. Therefore, this study presented a novel method of utilizing flexible energy storage devices for highly accurate and self-adaptive neuromorphic computational networks.

## METHODS

### Preparation of the few-layer Ti_3_C_2_T_x_ MXene

The 3 g of Ti_3_AlC_2_ MAX powder was added to 4.8 g of LiF. This mixture was added to 60 mL of 9 M of HCl at room temperature. Next, the mixed solution was magnetically stirred for 48 h to obtain multilayer Ti_3_C_2_T*_x_* powders. To peel off a few-layered Ti_3_C_2_T*_x_*, the obtained powders were dissolved in 75 mL of deionized water and centrifuged at 3500 rpm for 10 min. The procedures were repeated three times and the supernatant containing Ti_3_C_2_T*_x_* MXene flakes was collected each time for film fabrication.

### Fabrication of the FMES system

First, the electrodes of the supercapacitors were made on the polyethylene terephthalate (PET) substrate using a standard photolithography-thermal evaporation-stripping process, which was the Au film. Next, the MXene film was applied on the electrode surface with brushes. Finally, the H_2_SO_4_/PVA gel electrolyte (Supplementary Note 9) was evenly spread over the surface of the device.

## Supplementary Material

nwac158_Supplemental_File

## References

[1] Keene S-T , Lubrano C, Kazemzadeh S et al. A biohybrid synapse with neurotransmitter-mediated plasticity. Nat Mater 2020; 19: 969–73. 10.1038/s41563-020-0703-y 32541935

[2] Wang S , Wang C-Y, Wang P-F et al. Networking retinomorphic sensor with memristive crossbar for brain-inspired visual perception. Natl Sci Rev 2021; 8: nwaa172. 10.1093/nsr/nwaa172 34691573 PMC8288371

[3] Zhou F , Chai Y. Near-sensor and in-sensor computing. Nat Electron 2020; 3: 664–71. 10.1038/s41928-020-00501-9

[4] Zhou F-C , Zhou Z, Chen J-W et al. Optoelectronic resistive random access memory for neuromorphic vision sensors. Nat Nanotechnol 2019; 14: 776–82. 10.1038/s41565-019-0501-3 31308498

[5] Liao F-Y , Zhou Z, Kim B-J et al. Bioinspired in-sensor visual adaptation for accurate perception. Nat Electron 2022; 5: 84–91. 10.1038/s41928-022-00713-1

[6] He Y-L , Nie S, Liu R et al. Spatiotemporal information processing emulated by multiterminal neuro-transistor networks. Adv Mater 2019; 31: 1900903. 10.1002/adma.201900903 30957923

[7] Pan C , Wang C-Y, Liang S-J et al. Reconfigurable logic and neuromorphic circuits based on electrically tunable two-dimensional homojunctions. Nat Electron 2020; 3: 383–90. 10.1038/s41928-020-0433-9

[8] Lanza M , Sebastian A, Lu W-D et al. Memristive technologies for data storage, computation, encryption, and radio-frequency communication. Science 2022; 376: 6597. 10.1126/science.abj9979 35653464

[9] Zhu L-Q , Wan C-J, Guo L-Q et al. Artificial synapse network on inorganic proton conductor for neuromorphic systems. Nat Commun 2014; 5: 3158. 10.1038/ncomms4158 24452193

[10] Lai Q-X , Zhang L, Li Z-Y et al. Ionic/electronic hybrid materials integrated in a synaptic transistor with signal processing and learning functions. Adv Mater 2020; 22: 2448–53. 10.1002/adma.201000282 20446309

[11] Ahmed T , Tahir M, Low M-X et al. Fully light-controlled memory and neuromorphic computation in layered black phosphorus. Adv Mater 2021; 33: 2004207. 10.1002/adma.202004207 33205523

[12] Yang J-H , Xia Q-F. Battery-like artificial synapses. Nat Mater 2017; 16: 396–7. 10.1038/nmat4870 28272501

[13] Burgt Y-V , Lubberman E, Fuller E et al. A non-volatile organic electrochemical device as a low-voltage artificial synapse for neuromorphic computing. Nat Mater 2017; 16: 414–8. 10.1038/nmat4856 28218920

[14] Wei H-H , Shi R-C, Sun L et al. Mimicking efferent nerves using a graphdiyne-based artificial synapse with multiple ion diffusion dynamics. Nat Commun 2021; 12: 1068. 10.1038/s41467-021-21319-9 33594066 PMC7886898

[15] Rivnay J , Inal S, Salleo A et al. Organic electrochemical transistors. Nat Rev Mater 2018; 3: 17086. 10.1038/natrevmats.2017.86

[16] Zhao S-F , Ran W-H, Wang L-L et al. Interlocked MXene/rGO aerogel with excellent mechanical stability for a health-monitoring device. J Semicond 2022; 43: 082601.

[17] Merolla P-A , Arthur J-V, Icaza R-A et al. A million spiking-neuron integrated circuit with a scalable communication network and interface. Science 2014; 345: 668–73. 10.1126/science.1254642 25104385

[18] Wei H-H , Yu H-Y, Gong J-D et al. Redox MXene artificial synapse with bidirectional plasticity and hypersensitive responsibility. Adv Funct Mater 2021; 31: 2007232. 10.1002/adfm.202007232

[19] Kuzum D-G , Yu S-M, Wong H-S-P. Synaptic electronics: materials, devices and applications. Nanotechnology 2013; 24: 382001. 10.1088/0957-4484/24/38/382001 23999572

[20] Wang C-Y , Liang S-J, Wang S et al. Gate-tunable van der Waals heterostructure for reconfigurable neural network vision sensor. Sci Adv 2020; 6: eaba6173. 10.1126/sciadv.aba6173 32637614 PMC7314516

[21] Wan C-J , Cai P-Q, Guo X-T et al. An artificial sensory neuron with visual-haptic fusion. Nat Commun 2020; 11: 4602. 10.1038/s41467-020-18375-y 32929071 PMC7490423

[22] Seo S-H , Kang B-S, Lee J-J et al. Artificial van der Waals hybrid synapse and its application to acoustic pattern recognition. Nat Commun 2020; 11: 3936. 10.1038/s41467-020-17849-3 32769980 PMC7414205

[23] Zhang X-M , Zhuo Y, Luo Q et al. An artificial spiking afferent nerve based on Mott memristors for neurorobotics. Nat Commun 2020; 11: 51. 10.1038/s41467-019-13827-6 31896758 PMC6940364

[24] Choi Y-S , Oh S-Y, Qian C et al. Vertical organic synapse expandable to 3D crossbar array. Nat Commun 2020; 11: 4595. 10.1038/s41467-020-17850-w 32929064 PMC7490352

[25] Yu S-M . Neuro-inspired computing with emerging nonvolatile memory. Proc IEEE 2018; 106: 260–85. 10.1109/JPROC.2018.2790840

[26] Kim M-K , Lee J-S. Ferroelectric analog synaptic transistors. Nano Lett 2019; 19: 2044–50. 10.1021/acs.nanolett.9b00180 30698976

[27] Zhou J , Wan C, Zhu L et al. Synaptic behaviors mimicked in flexible oxide-based transistors on plastic substrates. IEEE Electron Device Lett 2013; 34: 1433–5. 10.1109/LED.2013.2280663

[28] Li X , Tang J, Zhang Q et al. Power-efficient neural network with artificial dendrites. Nat Nanotechnol 2020; 15: 776–82. 10.1038/s41565-020-0722-5 32601451

[29] Sun J , Oh S, Choi Y-S et al. Optoelectronic synapse based on IGZO-Alkylated graphene oxide hybrid structure. Adv Funct Mater 2018; 28: 1804397. 10.1002/adfm.201804397

[30] Yu J-R , Yang X-X, Gao G-Y et al. Bioinspired mechano-photonic artificial synapse based on graphene/MoS_2_ heterostructure. Sci Adv 2021; 7:eabd9117. 10.1126/sciadv.abd9117 33731346 PMC7968845

[31] Li C , Belkin D, Li Y-N et al. Efficient and self-adaptive *in-situ* learning in multilayer memristor neural networks. Nat Commun 2018; 9: 2385. 10.1038/s41467-018-04484-2 29921923 PMC6008303

[32] Zhou T , Lin X, Wu J et al. Large-scale neuromorphic optoelectronic computing with a reconfigurable diffractive processing unit. Nat Photon 2021; 15: 367–73. 10.1038/s41566-021-00796-w

