# Supplementary material for: Neuromorphic-computing-based adaptive learning using ion dynamics in flexible energy storage devices
Source: Natl Sci Rev. 2022 Aug 13;9(11):nwac158. doi: 10.1093/nsr/nwac158 (PMC9646995; doi:10.1093/nsr/nwac158)
Supplement: nwac158_Supplemental_File [file nwac158_supplemental_file.pdf]

Supplementary Data

Neuromorphic-computing-based adaptive learning using ion dynamics in flexible energy storage devices

Shufang Zhao[1,‡], Wenhao Ran[1,‡], Zheng Lou[1], Linlin Li[1], Swapnadeep Poddar[2], Lili Wang[1,*], Zhiyong Fan[2,*] and Guozhen Shen[3,*]

[1]State Key Laboratory for Superlattices and Microstructures, Institute of Semiconductors, Chinese Academy of Sciences & Center of Materials Science and Optoelectronic Engineering, University of Chinese Academy of Sciences, Beijing 100083, China;

[2]Department of Electronic & Computer Engineering, The Hong Kong University of Science and Technology, Hong Kong, China;

[3]School of Integrated Circuits and Electronics, Beijing Institute of Technology, Beijing 100081, China

***Corresponding authors**. E-mails: liliwang@semi.ac.cn; eezfan@ust.hk; gzshen@bit.edu.cn

[‡]Equally contributed to this work.

This PDF file includes:

Figs. S1 to S25

Note 1-9

Supplementary Note 1. Synaptic plasticity of the FMES device. Supplementary Fig. 6 shows the current-voltage (C-V) characteristics at different sweep rates of the FMES device. With the voltage sweeping from 0 to 0.8 V, a clear clockwise loop can be observed with a large negative current at voltage = 0 V, revealing a nonvolatile change of output voltage which is vital for the emulation of synaptic function. In addition, the C-V curves of the device are also different at different sweep rates. This phenomenon indicates the memorizing capability and synaptic ability of our device can be modulated by the amplitude, duration and frequency of input pulses. Thus, it is essential to study the impact of input pulse amplitude, duration and frequency on the storage statues of the FMES device. In order to further explore the device properties under different pulses stimulating the artificial synapse, varying excitatory pre-synaptic current (I_{spike}) with different amplitudes (30 mA to -30 mA, Supplementary Fig. 7a) and width (100 ms to 500 ms, Supplementary Fig. 7b) were applied to the artificial synapse. With the increase in the intensity of the I_{spike} and the broadening of the pulse width, the output post-synaptic voltage (PSV) gradually enhances, which demonstrates more robust synaptic plasticity. According to the aforementioned working principle, the rise in voltage and subsequent decline of the excitatory post-synaptic voltage (EPSV) depend on the adsorption and desorption of two ions on the MXene surface. The higher frequency and stronger current allows the device to receive more charge, which trigger accelerates the adsorption of more ions, thereby rendering a higher voltage. As an important manifestation of the synaptic plasticity, short-term plasticity (STP) plays a pivotal role in the realization of

advanced learning and memorization. The most typical behavior is the paired-pulse facilitation (PPF), in which the amplitude of the EPSV triggered by the second excitatory pulse (A_2) is apparently larger than the first excitatory pulse (A_1). It is obvious, the two excitatory stimulations further amplify the PSV, resulting in $A_2>A_1$, indicating that the device has clear PPF behavior. Subsequently, interval time of the two consecutive current pulses is changed, and the PPF index is calculated by:

$$PPF=100\% \times A_2/A_1$$

whose results are shown in Supplementary Fig. 7c. As can be seen from the results, with increase of the interval time (Δt), the PPF index gradually decreases and finally decays to 100% nearly, implying that the second pulse is not impacted by the previous pulse at this instant. This behavior is similar to biological synaptic behavior. For electrically stimulated PPF, a shorter Δt disallows the ions adsorbed on the MXene to be completely desorbed before applying the second pulse. Consequently, when the second pulse is applied, the ions adsorbed on the MXene will further increase, resulting in a higher PPF index. Otherwise, a longer Δt allows enough time for the ions adsorbed on the MXene to return to the initial state, leading to a lower PPF index.

In order to further examine the multi-spiking effect of the FMES device, five successive voltage pulses with amplitude of 0.1 mA, width of 5 ms and different frequency (from 2 Hz to 100 Hz), were applied to the artificial synapse, whose corresponding response results are shown in Supplementary Fig. 8. We extracted the spike voltage of each stimulus at different frequencies and plotted in Supplementary

Fig. 7d. Apparently, when the frequency is lower, such as 2 Hz, the peak voltage did not change with the number of pulses. On the contrary, when the frequency is higher, such as 25 Hz, the peak voltage gradually increases as the number of pulses increases, which is akin to the advanced synaptic function in synaptic plasticity, namely, the 'learning-forgetting-relearning' process. In the human brain, the law of leaning and forgetting is called Ebbinghaus forgetting curve. Supplementary Fig. 8 depicts the simulation results of our device for this behavior, including 3 times of learning-forgetting process through continuously application and removal of current pulses. After 29 excitatory current spikes, the PSV is significantly increased, and then it merely takes 14 excitatory current spikes to reach the same PSV after a period of attenuation. Repeating the above process again, only 6 excitatory current spikes can reach the initial memory level. And, it is evident that the level of residual memory (PSV) is higher and higher after each forgetting, implying that a transition from short-term memory (STM) to long term memory (LTM) is realized.

Supplementary Note 2. The movement and accumulation of cations. For the high-resistance state (Fig. 1d, left), the anions tend to move toward the direction of the higher potential during the application of I_{spike}, while the cations tend to move toward the direction of the lower potential. The electrolyte can only assist in ionic transport but not in the movement of electrons. Therefore, anions and cations are firmly bound to the left and right electrode positions, respectively. For the low-resistance state (Fig. 1d, right), the movement speed of cations and anions in the capacitor decreases significantly compared to that in the high-resistance state during

the application of I_{spike}. The number of ions accumulated at both ends of the capacitor during the same stimulation time decreases, resulting in a net decrease in the stored voltage. After removing I_{spike}, the agility of the ions increases further, when compared to that in the high-resistance state, causing the excitatory postsynaptic voltage (EPSV) to decay faster. In the FMES artificial synaptic device, the synaptic weights are proportional to the number of cations and anions or the conductivity of a synapse. Therefore, resistance-controlled accumulation and dissipation of cations and anions within an active synaptic device area can imitate the linear variations in the synaptic weights during potentiation and depression, respectively.

Supplementary Note 3. Durability and stability of the devices. The long-term working ability of synaptic devices is very important for neuromorphic computing. We randomly select six voltage states for retention characterization. We first randomly programmed the postsynaptic voltage of the device to the corresponding position with a 1 mA, 500 Hz pulsed current. Then we removed the pulsed current and continuously monitored the device for postsynaptic voltage changes. As shown in Supplementary Fig. 10a, the variation of each voltage state is almost negligible over a 1500 s time scale, which confirms that the device exhibits better retention of programmed postsynaptic voltage. Endurance is another important device criterion, especially for the inference process in neural networks, which determines the long-term working capability of artificial synaptic devices. Supplementary Fig. 10b shows the I-V loops for 95 cycles for two sweep rates. The results show that at the 100 mV/s and 300 mV/s sweep rates, the I-V loops for 95 cycles are basically the same, which indicates

that the device exhibits good endurance. For further evaluation of the device endurance, we obtain potentiation and depression behaviors of the artificial synapses by alternating a series of 300 identical pulses (Supplementary Fig. 10c). Furthermore, we extracted and enumerated the highest postsynaptic voltage (V_{max}), and Supplementary Fig. 10d, shows the lowest postsynaptic voltage (V_{min}) in each of the above 100 cycle results. The distribution results show that the rate of change over 100 cycles in V_{max} is small, i.e., lower than 10%. Although the change ratio of V_{min} is large, its actual amplitude change is not high. The test results confirm that in 100 consecutive cycles, there is almost no obvious change in the postsynaptic voltage, which indicates that the device has good endurance during weight updates. In addition, in an in-memory computing array, the differences between devices are vital. To verify the variability from device to device, we fabricated the array shown in Supplementary Fig. 11a. It consists of 25 devices, which can perform 25 multiplications and 1 addition simultaneously. We applied 300 current pulses to each device in the array to test its potentiation and depression behaviors, the results of which are shown in Supplementary Fig. 11b. The results show that the 25 devices' postsynaptic voltages varied from 0.05 to 0.6 V when stimulated by 300 consecutive current pulses. Supplementary Fig. 11c statistically compares the highest postsynaptic voltage (V_{max}) and the lowest postsynaptic voltage (V_{min}) of the 25 devices in the above test results. The statistical results show that the V_{max} and V_{min} fluctuation between different devices is insignificant. Furthermore, we also measure the I-V characteristics in sweep cycles of 0 to 0.8 V with a 100 mW·s^{-1} sweep rate of the 25 devices, the results

of which are shown in Supplementary Fig. 11d. Several curves are almost completely coincident with very small errors. This shows that there is less variation from device to device. These measured results all illustrate that our device-to-device variability is less.

Supplementary Note 4. Autocorrelation analysis. At this stage, there is no strict definition of long-term memory and short-term memory of synaptic devices. In the field of statistics, long-term memory and short-term memory have strict definitions, that is, for a second-order stationary one-dimensional time series, Long term memory [r(k)] is defined by its autocorrelation function (autocovariance function) such that

$$\sum_{k=-\infty}^{\infty} r(k) = \infty$$

On the contrary, if the synaptic devices have short-term memory, r(k) is defined as

$$0 < \sum_{k=-\infty}^{\infty} r(k) < \infty$$

Therefore, we analyzed the memory capacitance of the FMES synaptic system from the perspective of autocorrelation. We first solved the autocorrelation function of the FMES synaptic system, which is shown in Supplementary Fig. 12a, left). We select all the post-synaptic voltage (PSV) curves tested (some examples shown in Fig. 2e) under the excitatory current with same amplitude (0.1 mA) and different width at different regulating resistance for autocorrelation analysis. Through the formula,

$$R_v(\tau) = \lim_{T\to\infty} \frac{1}{T} \int_{-T/2}^{T/2} V(t)V(t+\tau)d\tau$$

we can obtain the autocorrelation function corresponding to all PSV curves and normalize them (some examples are shown in Supplementary Fig. 12a, right).

Obviously, since $R_v(\tau)$ is constantly decays, the integral of $R_v(\tau)$ must be less than ∞. Therefore, theoretically, the FMES synaptic system has short-term memory and no long-term memory. However, if the integral of $R_v(\tau)$ is to be infinite, unless the damping of the synaptic system is 0, that is there is no energy loss, which could not be achieved by all synaptic systems currently studied. Although, in theory, the FMES synaptic system has only short-term memory, as the regulating resistance increases, the curve gradually flattens (Supplementary Fig. 12b), which indicates that the integral of $R_v(\tau)$ increases, indication the enhancement of memory. This result shows that changing the regulating resistance will affect the memory of the FMES synaptic system. In order to clearly demonstrate the influence of the regulating resistance on the memory of the FMES synaptic system, we use Gaussian function to fit all the autocorrelation functions. If the standard deviation of the Gaussian function is larger, the curve of the autocorrelation function will be flatter, which means the memory capacity of the FMES synaptic system will be stronger and the system would driftcloser it will be to long-term memory.

Supplementary Note 5. The neural network based on FMES synaptic system simulations.

Training and testing dataset. To perform the simulations of neuromorphic computation based on the FMES synaptic system, we used the MNIST datasets of handwritten digits as the learning and testing samples, which was downloaded from online available resource (webpage: http://yann.lecun.com/exdb/mnist/). The dataset includes 60,000 training images and 10,000 testing images, which is shown in Fig

S19a.

Weight update for FMES synaptic system. For our FMES synaptic system, we use excitatory current spikes and inhibitory current spikes to achieve long-term potentiation (LTP) and long-term depression (LTD). In the experiment, the excitatory current spike is set to I_{spike} = 1 mA, t_{spike} = 0.001s and the inhibitory current is set to I_{spike} = -1 mA, t_{spike} = 0.001s. We find that the change of synaptic weight in the neural network is based on the LTP and LTD characteristics of the FMES synaptic system, which is related to the regulating resistance. Therefore, we examine the LTP and LTD of the FMES synaptic system under different regulating resistance (500 Ω, 2000 Ω, 8000 Ω and the open state).

To define the weight updating rule of FMES synaptic system under different regulating resistance, we extracted the stable post-synaptic voltage (PSV) after each current spike as the weight to get the LTP and LTD curves. We use the following formula to fit the LTP and LTD curves under different regulating resistance, so as to obtain the nonlinearity (NL) and the weight change of LTP and LTD during the update process:

$$V_{i+1} = V_i + \Delta V_p = V_i + \alpha_p e^{-\beta_p \frac{V_i - V_{min}}{V_{max} - V_{min}}}$$

$$V_{i+1} = V_i + \Delta V_d = V_i + \alpha_d e^{-\beta_d \frac{V_{max} - V_i}{V_{max} - V_{min}}}$$

where, V_i and V_{i+1} are the PSV of ith and $(i+1)^{th}$ spike, V_{max} and V_{min} are the minimum and maximum voltage, α_p and α_d are the differences in the voltage between two points on the potentiation and depression curves, β_p and β_d represent the NL of the potentiation and depression curves, respectively. Each regulating resistance

corresponds to a set of LTP/LTD fitting curves, representing a set of weight update rules.

To determine the direction of weight update, the current at output neurons of image m were transformed by a softmax algorithm, resulting in a set of output neuron signals ($f_{m,n}$, n = 0, 1, ……, 9). Comparing the label values ($k_{m,n}$) corresponding to the image m, we can obtain the delta value ($\delta_{m,n} = k_{m,n} - f_{m,n}$). If $\delta > 0$, the synaptic weight (W) and V are increase (W↑ = V↑), else, the W and V are depression (W↓ = V↓). The weight changes (ΔW) and voltage changes (ΔV) are determined by the above fitting formulas.

FMES-based neural network. During the process, each neuron in the input layer received the corresponding value of the pixel in the image, converted into a conductance state, assigned it to the input vector ($X_{\text{pixel m}}$), and further converted into 10 output values (Σ_n) through the weight matrix ($W_{m,n}$) to be used by the neurons. Because the weight value is the PSV, it can be used for matrix operation with conductance. Here, Σ_n was converted to the output vector (Y_n), which is the activation function. A total of 60,000 images in the MNIST dataset were used to train the FMES-based neural network, and 10,000 test images not provided during training were used to calculate the recognition accuracy of 10 digits. During the training process, the PSV of the proposed FMES device ($W_{m,n}$) was used to update the synaptic weights of the FMES-based neural network.

Supplementary Note 6. The working principle of the conceptual hardware neural network comprising the FMES artificial synaptic devices.

The hardware neural network contains multiple processing units. NMOS1 functions as a gate to determine the operation of the FMES system (weight update), and C represents the FMES. NMOS2 and C form the FMES system. HSPICE was used to verify the designed system architecture. When the gate voltage of NMOS2 increased, the LTP and LTD characteristics of the FMES system changed, proving that the proposed design was feasible. Each processing unit is composed of an FMES synaptic system and one NMOS to complete the processing and storage process, 2T1C architecture. MOS1 is a strobe gate, used for current pulse input FMES synaptic system, which can change the PSV of FMES and realize weight adjustment. FMES synaptic system consists of MOS2 and C, where MOS2 acts as a regulating resistance and C represents FMES. During the experiment, the PSV of the FMES synaptic system is low and thus the voltage across the source and drain of MOS2 is low, resulting in MOS2 operating in the linear region. Since MOS2 operates in the linear region, we can adjust the resistance of MOS2 by changing the gate voltage Vg of MOS2 to realize the adjustment weight change (ΔW) of the FMES synaptic system and change the learning rate.

Supplementary Note 7. Adaptive mechanism. Recently, there have been many research results using artificial synaptic devices to construct artificial neural networks for neuromorphic calculations. However, most of research has a problem, that is the neural network trained through the training set can only have perfect recognition results for specific types of test data. If the type of test data changes, the stored weights of the synaptic devices in the artificial neural network must be erased and

then retrained to adapt to the new type of data. For example, dataset 1 and dataset 2 are composed of dogs and cats, respectively. The artificial neural network trained in dataset 1 can effectively identify dogs. If we want the artificial neural network to be able to effectively identify cats, we must retrain the artificial neural network to adapt to the dataset 2. The whole process consumes a lot of energy and time. Based on this, we put forward the concept of adaptive learning, which is input some of the pictures in the dataset 2 into the above-mentioned trained artificial neural network, the synaptic devices in the network can automatically adjust the weight to adapt to classify and recognize dataset 2. At this point, dataset 2 can be effectively identified and classified. The adaptive method can not only save the time spent on retraining the artificial neural network, but also reduce power consumption. Since our FMES synaptic device includes a MXene-supercapacitor for weight storage and an adjustment resistance for synaptic performance adjustment, the complex synaptic performance and working mode can perfectly realize the above adaptive method. The main processes are as follows: First, after finishing training, the weight of FMES synaptic device is changed and stored. This is mainly because the MXene-supercapacitor in the FMES synaptic device stores charges during the learning and training process, which causes the voltage of the FMES synaptic system to change. At this time, the resistance value of the regulation resistance is adjusted to the maximum, so that the charge stored on the MXene-supercapacitor will hardly be lost due to the internal self-discharge of the FMES synaptic system, ensuring that the weight (voltage) of the FMES synaptic system will hardly change. Then, we extract a

part of the image from the dataset to be identified and convert the pixel points into a resistance value, wherein the pixels with a small value are converted into a small resistance value and the pixels with a large value are converted into a large resistance value. Finally, we set the resistance value of the regulation resistance in the FMES synaptic system to the converted resistance value, and use the self-discharge in the FMES synaptic system within a short period of time to consume the energy stored in the MXene-supercapacitor to realize the FMES synaptic system weight adjustment. After many cycles of the last step, the weight of the entire artificial neural network is adjusted to adapt to the dataset to be identified. Consequently, the entire adaptive weight adjustment process is completed.

Supplementary Note 8. Device performance for neuromorphic computing. We devised eight patterns as shown in Supplementary Fig. 25a, including ‘A’, ‘B’, ‘C’, ‘D’, ‘E’, ‘F’, ‘I’, ‘J’, where 1 represents low resistance and 0 represents high resistance. Each pattern is 5×5 in size. We first designed an algorithm using MATLAB to obtain the weight (voltage) of each unit in the weight matrix that identifies each letter. Then, the obtained weight voltage of the weight matrix was used to identify the letter ‘A’. We stimulated the postsynaptic voltages of 25 devices in the prepared 5×5 array to corresponding values by using a pulse current of 1mA, 500 Hz. This distribution is shown in the first column of Supplementary Fig. 25b. Finally, we connect the patterns of 8 letters to the matrix respectively to obtain the output currents, which are shown in the first column of Supplementary Fig. 25c. Following the same method, we stored the postsynaptic voltages of the weight matrix used to identify the

letters ‘B’, ‘C’, ‘D’, ‘E’, ‘F’, ‘I’, ‘J’, respectively and connect them to perform operations. Supplementary Fig. 25b shows the distribution of the postsynaptic voltages for the weight matrix used to identify the 8 letters stored in the 5×5 array. Supplementary Fig. 25c shows the output currents obtained by separately operating with the 8 letters after storing the weight voltage for identifying the arrangement of letters in a 5×5 array each time. Each row in Supplementary Fig. 25c represents the current output after 8 arrangements of letters are connected with 8 recognition matrices respectively. The results confirm that the maximum current value of each row appears on the diagonal of the diagram. This shows that image recognition can be achieved by setting the postsynaptic voltage of each unit in the 5×5 array to the value of the weight matrix for recognizing the corresponding letter obtained by the algorithm simulation. To clarify the result in Supplementary Fig. 25c, we introduce nonlinearity and process it with the softmax operation to obtain the probability distribution shown in Supplementary Fig. 25d. Again, the maximum probability occurs on the diagonal, indicating that the correct pattern can be recognized.

Supplementary Note 9. Fabrication of H_2SO_4/PVA gel electrolyte. In this procedure, 3.0 g PVA was dispersed into 30 mL of deionized water. The mixture was subsequently heated in an oil bath at 95 ℃ under magnetic stirring for 5 h to form a uniform colloidal solution. After cooling to room temperature, 1.7 mL of H_2SO_4 was added the aforementioned colloidal solution and magnetically stirred for 6 h until the solution is uniform. Finally, we obtained the H_2SO_4/PVA gel electrolyte.

Characterization and measurement: The synaptic performance of the fabricated

devices was measured by the CHI (760D) electrochemical work station. A TEM with an EDX analyzer (JEM-2010F) and an SEM (Zeiss Supra55 (VP)) were both employed to measure the sizes and morphologies of the product (MXene). The purity of the product was characterized with X-ray diffractometer (XRD, DMAX-RB).

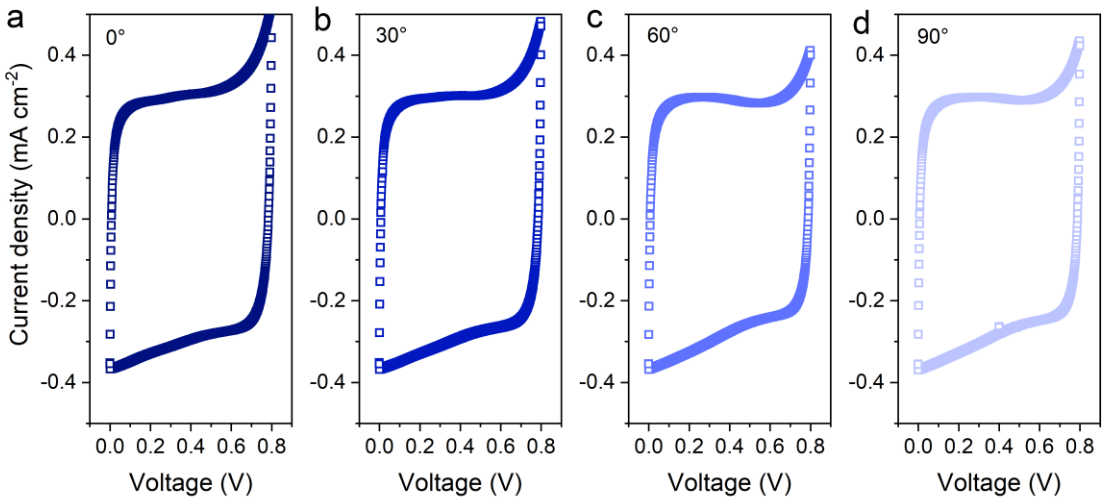

Supplementary Fig. 1 The C-V curves of the FMES device at different bent state: a 0 °, b 30 °, c 60 °, and d 90 °.

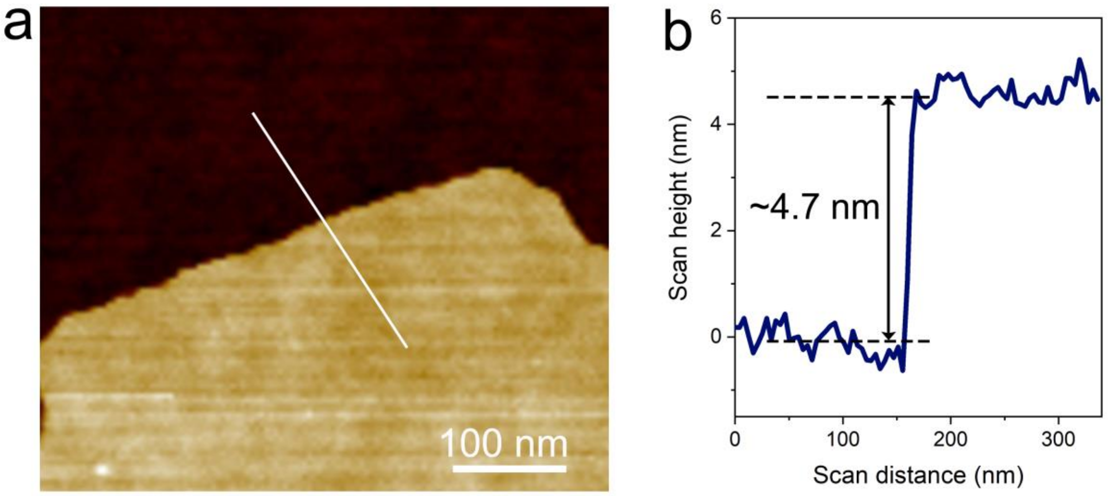

Supplementary Fig. 2. The Thickness characterization of the MXene. (a) The AFM image of the MXene, (b) The thickness of the MXene.

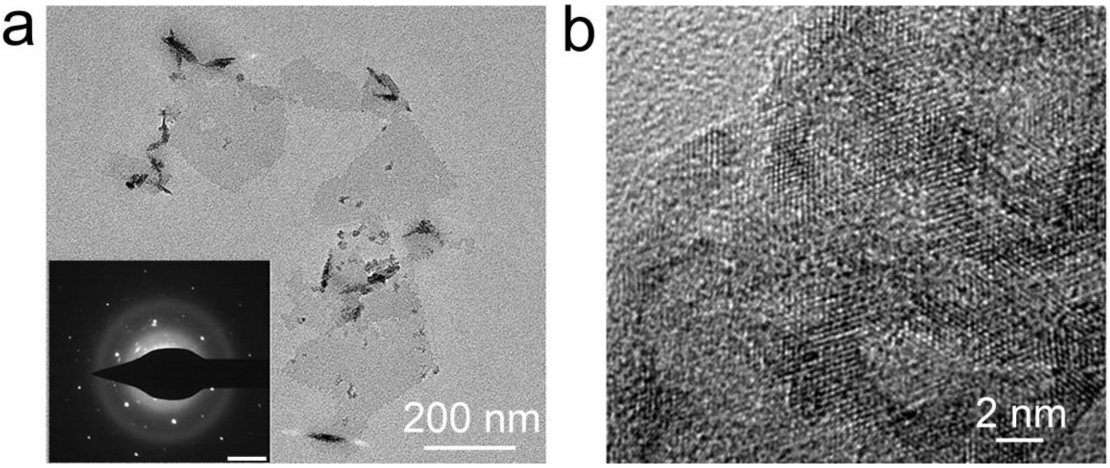

Supplementary Fig. 3 (a) The transmission electron microscope (TEM) image of MXene and (b) The high-resolution transmission electron microscope (HRTEM) image of MXene.

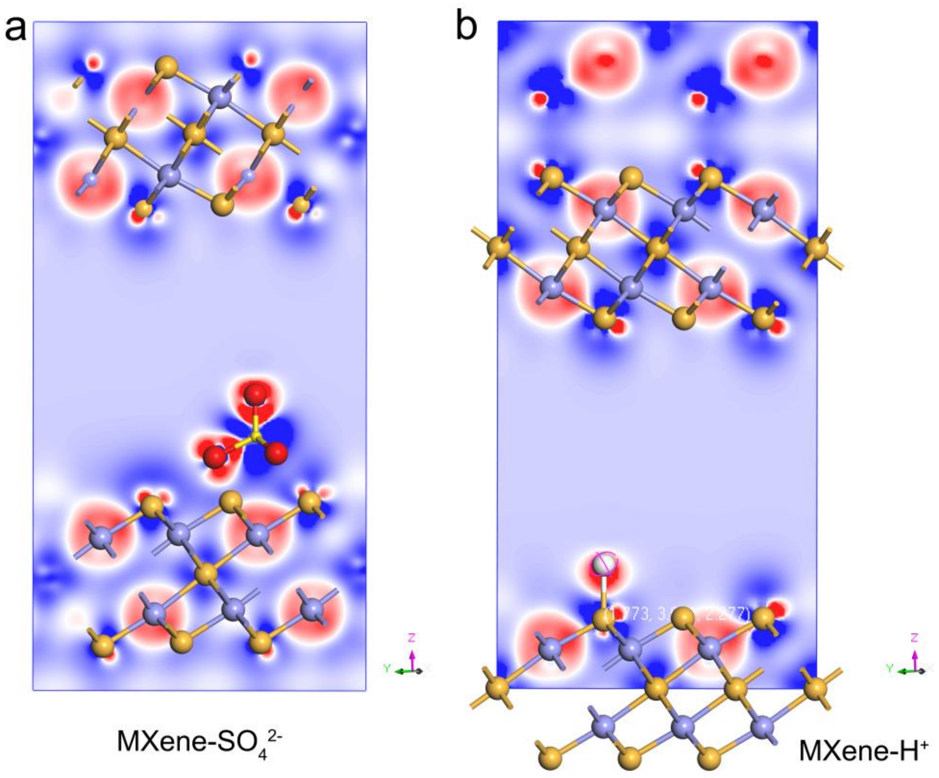

Supplementary Fig. 4 (a) The physical adsorption characteristics of H^+ and SO_4^{2-} on the surface of MXene. (b) Cross-sectional charge density different distribution of a SO_4^{2-} on the surface of MXene and b H^+ on the surface of MXene.

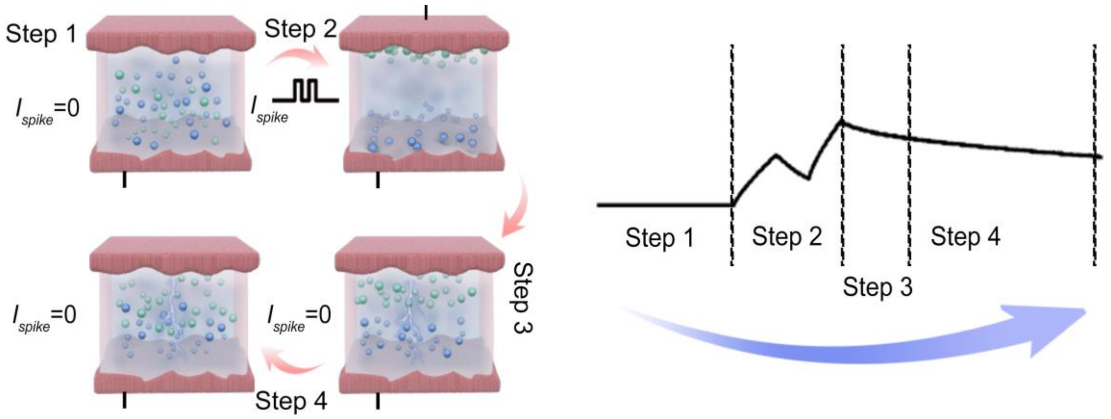

Supplementary Fig. 5 Schematic diagram of the working mechanism and process of FMES device.

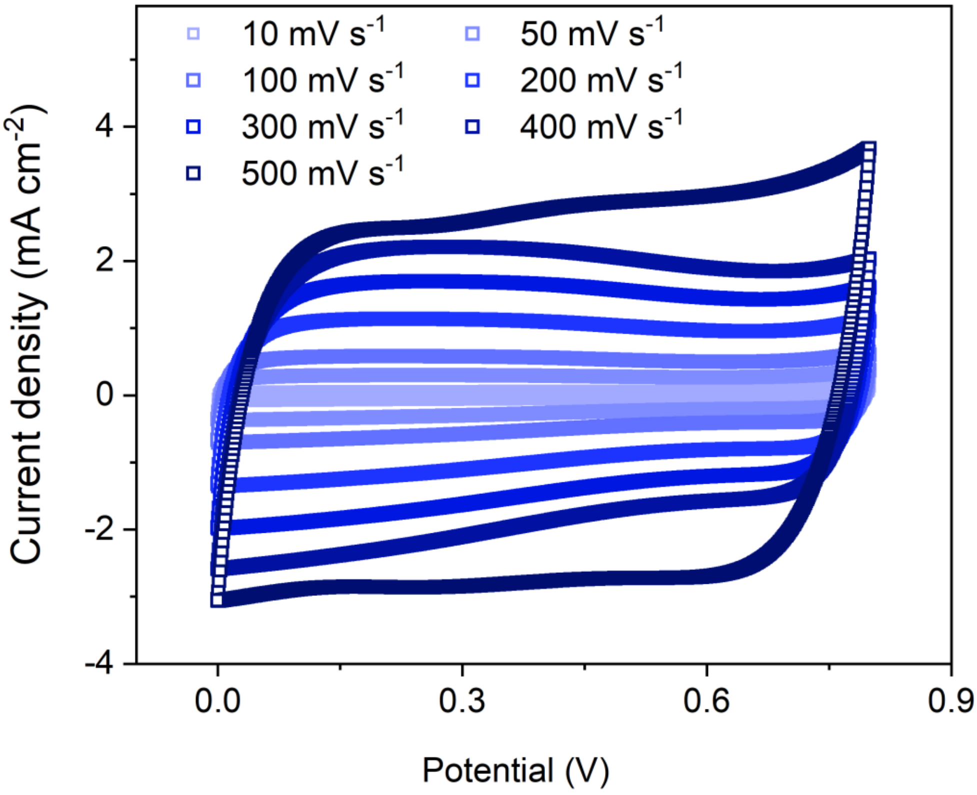

Supplementary Fig. 6 The C-V curves of the FMES device at different sweep rate, which indicating that FMES device has synaptic function.

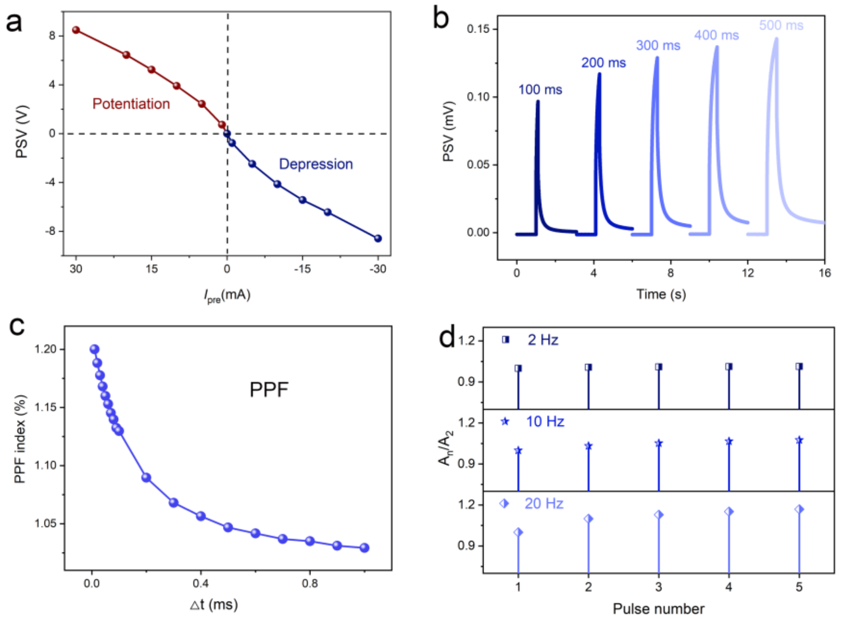

Supplementary Fig. 7 (a) The relationship between post-synaptic voltage (excitatory post-synaptic voltage (EPSV) and inhibitory post-synaptic voltage (IPSV)) and pre-synaptic current spike (I_{spike}) amplitudes under same spike time. (b) The impact of spike duration on EPSV under same current spike amplitudes. (c) The PPF index connects with time interval (Δt) of continuous current spike. (d) The EPSVs triggered by current pulse including five presynaptic pulse under different frequencies.

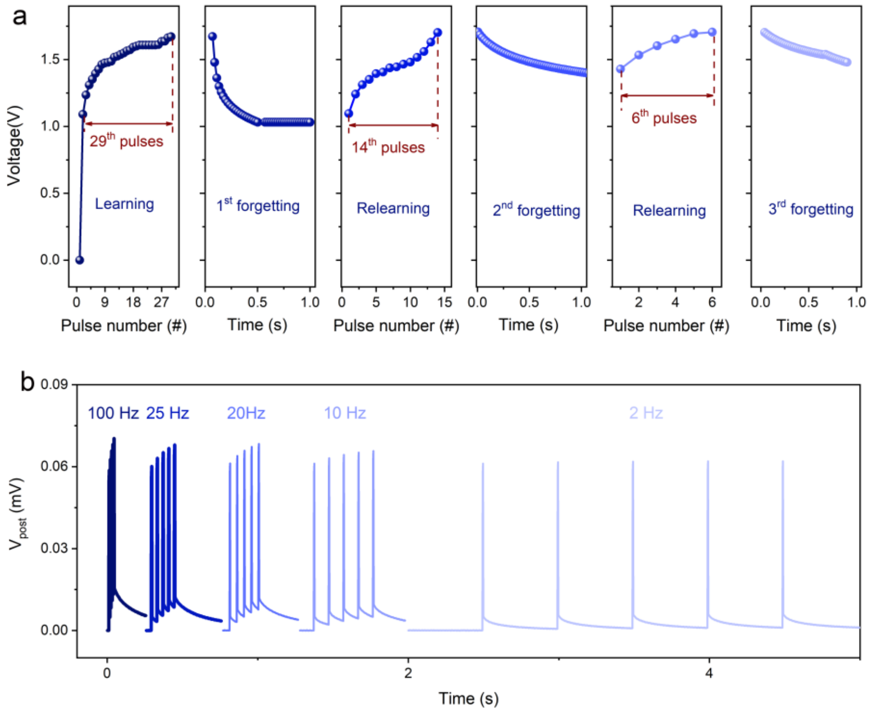

Supplementary Fig. 8 (a) Emulation of learning-experience behavior with electric current spike, which is similar to the learning-forgetting-relearning mechanism in the human brain. (b) Multi-spiking effect of the FMES device at different frequency.

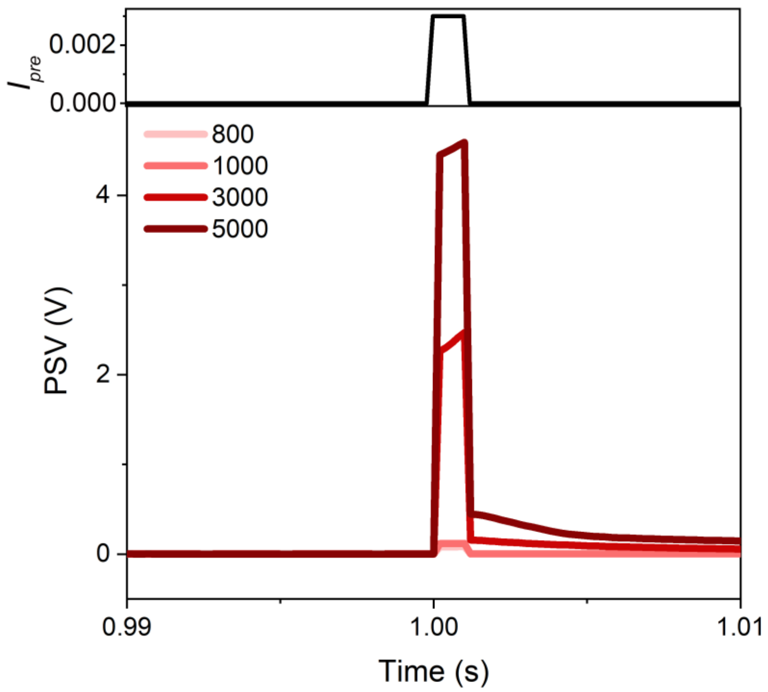

Supplementary Fig. 9 The change of PSV with resistance under the same I_{spike}.

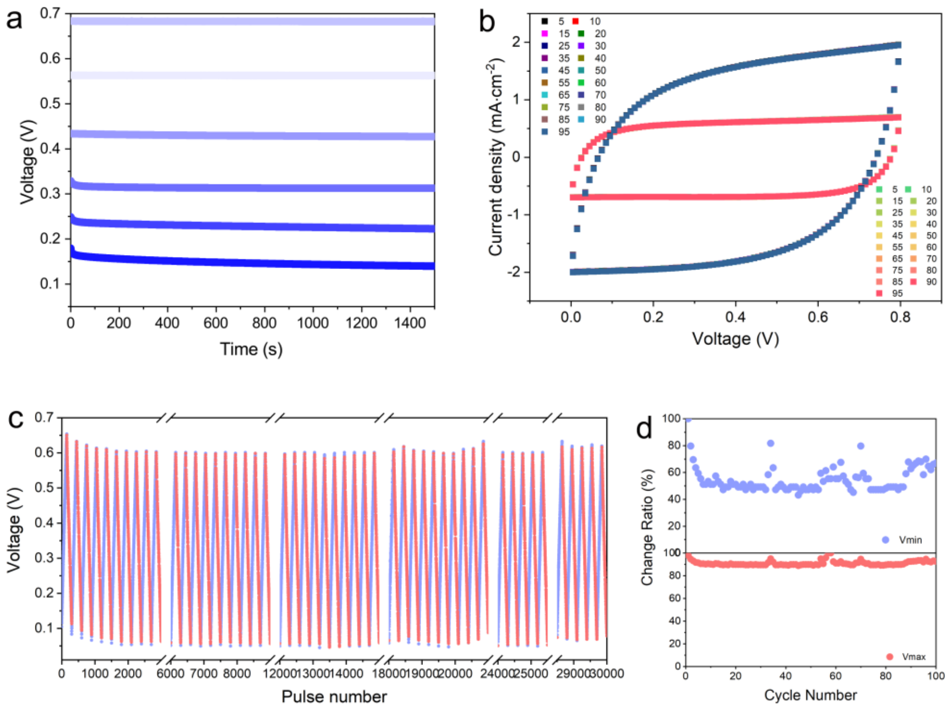

Supplementary Fig. 10 (a) Six voltage states randomly distributed in the switching range are selected for retention measurement. (b) I-V curves measured 95 times in sweep cycles of 0 to 0.8 V with 100 mW·s^{-1} and 300 mW·s^{-1} sweep rate. (c) Postsynaptic voltage monitored at 300 current pulses were continuously cycle 100 times, where the first 150 pulses in each cycle correspond to dynamic potentiation behaviors and the last 150 pulses correspond to dynamic inhibition behaviors in postsynaptic voltage. (d) Distribution of the V_{min} and V_{max} of 100 cycles.

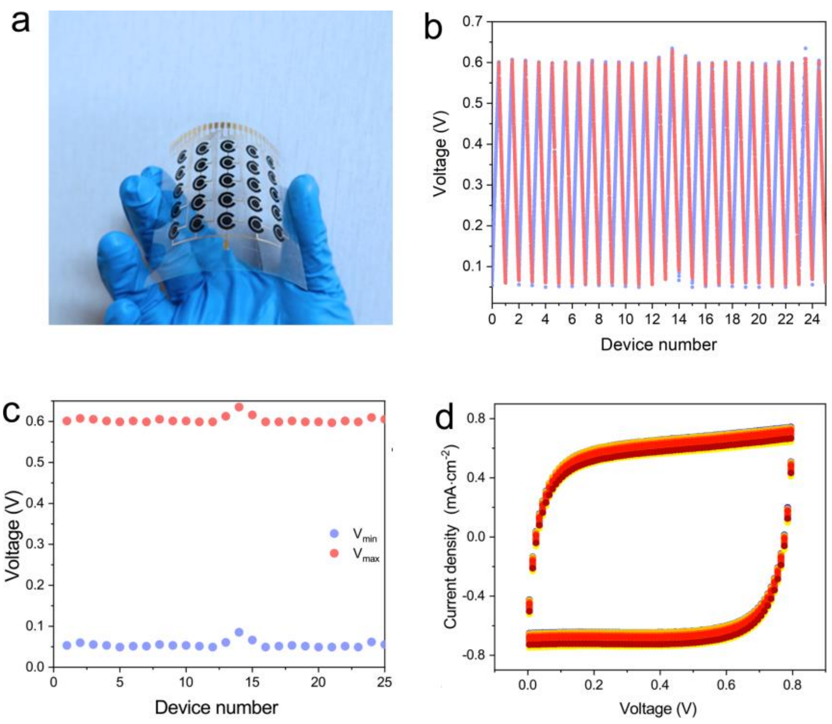

Supplementary Fig. 11 (a) Schematic diagram of 5×5 synaptic device array. (b) Postsynaptic voltage monitored at 300 current pulses of 25 devices from 5×5 array. (c) Distribution of the V_{max} and V_{min} of 25 devices from 5×5 array. (d) I-V curves measured in sweep cycles of 0 to 0.8 V with 100 mW·s^{-1} sweep rate of 25 devices.

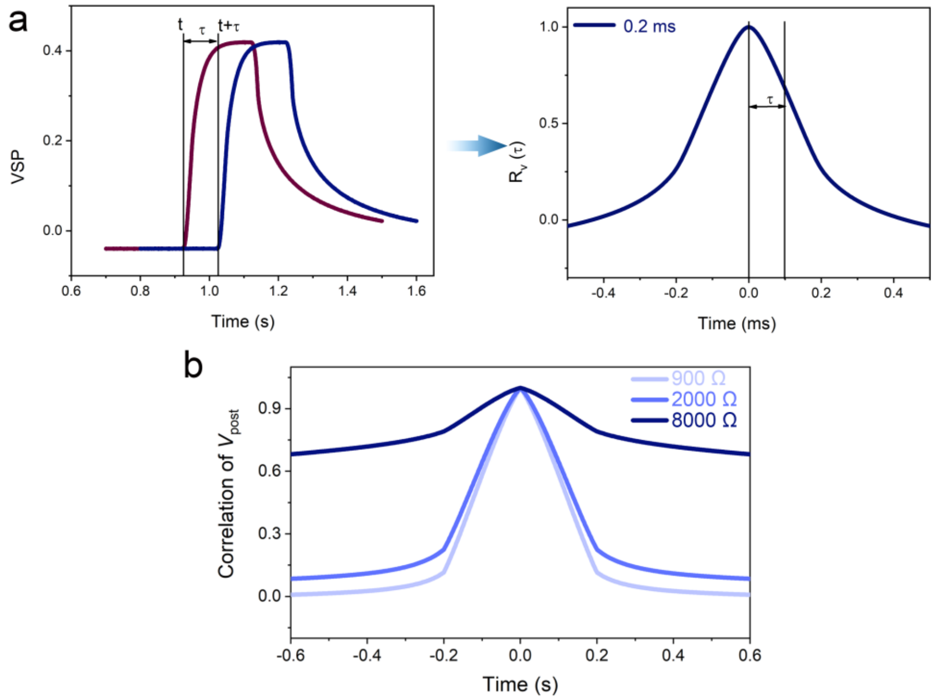

Supplementary Fig. 12 (a) The calculation method of autocorrelation function of the FMES synaptic system. (b) The change of autocorrelation function with resistance under the same I_{spike}.

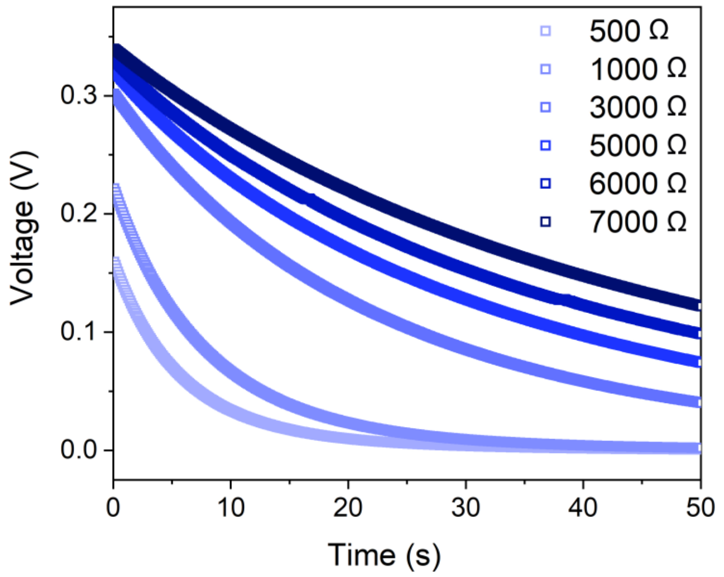

Supplementary Fig. 13 The long-term plasticity (LTP) changes with resistance after a long-term presynaptic current stimulation.

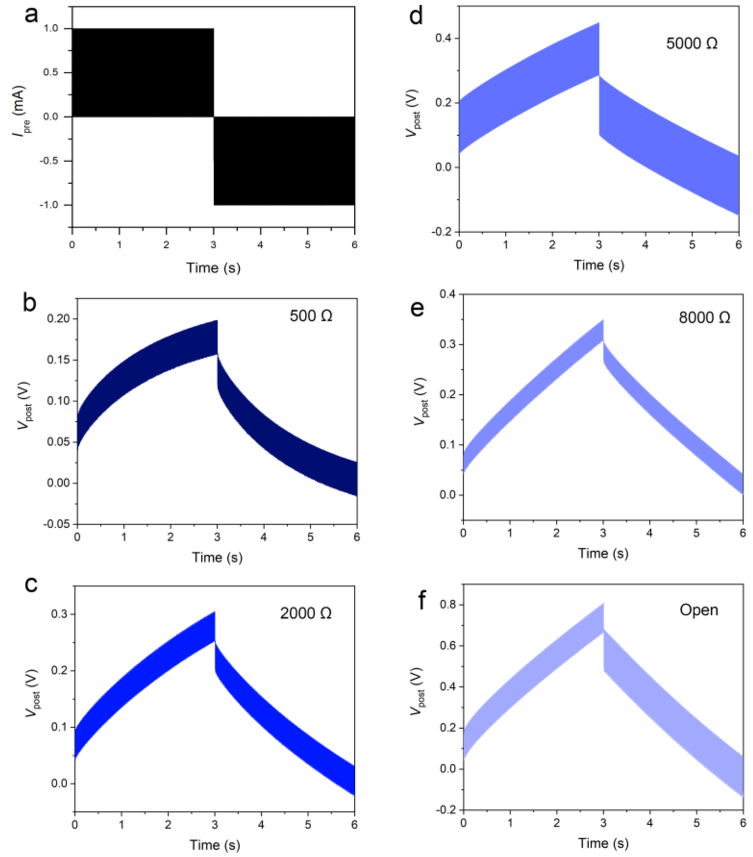

Supplementary Fig. 14 The LTP/LTD characteristics of FMES artificial synaptic devices measured by the application of 150 potentiation pulses (1 mA) and 150 depression pulses (-1 mA) under various resistance states: (a) current pulses-controlled, (b) 500 Ω, (c) 2000 Ω, (d) 5000 Ω, (e) 8000 Ω, and (f) the open state.

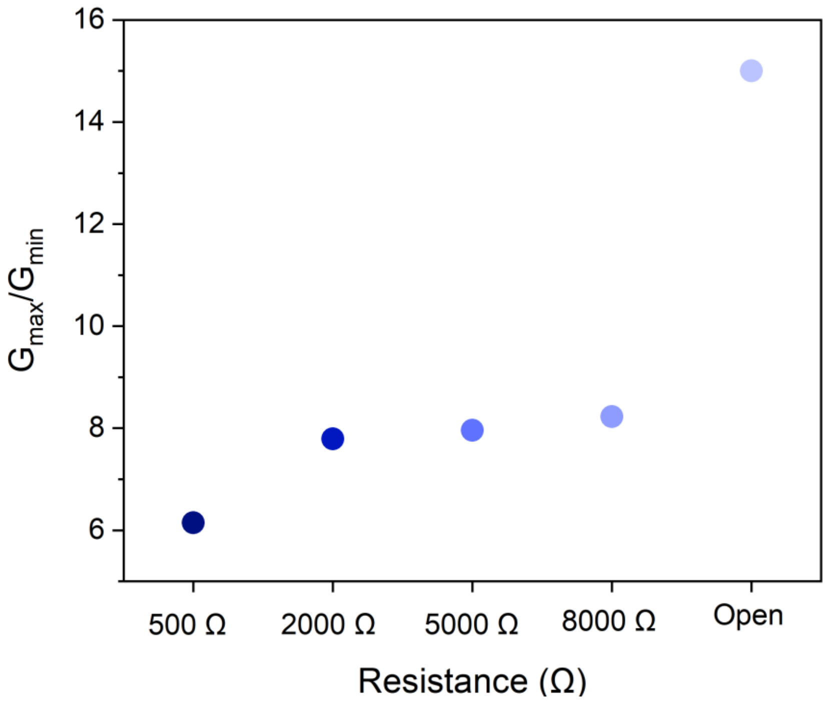

Supplementary Fig. 15 The dynamic range (W_{max}/W_{min}) values of the FMES artificial synaptic devices under 500, 1000, 8000 Ω and OPEN state.

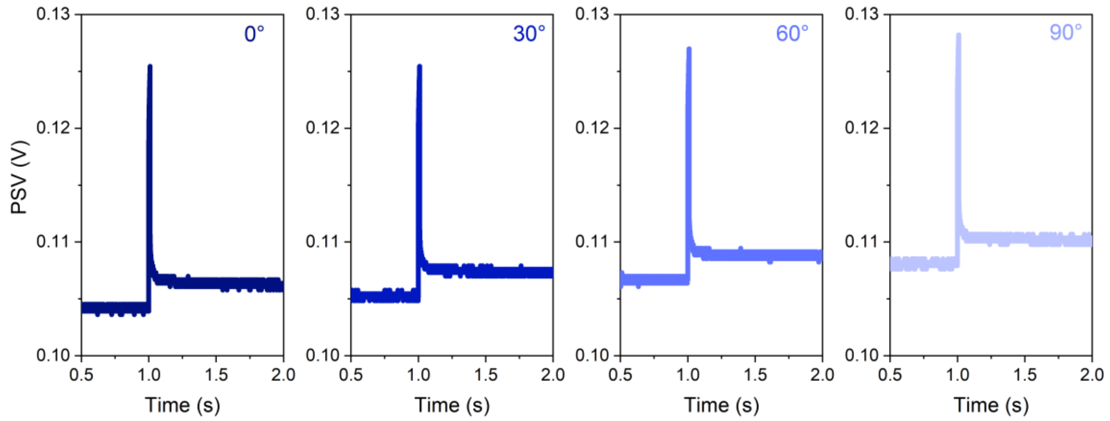

Supplementary Fig. 16 Synaptic plasticity of FMES synaptic system under different bending angles.

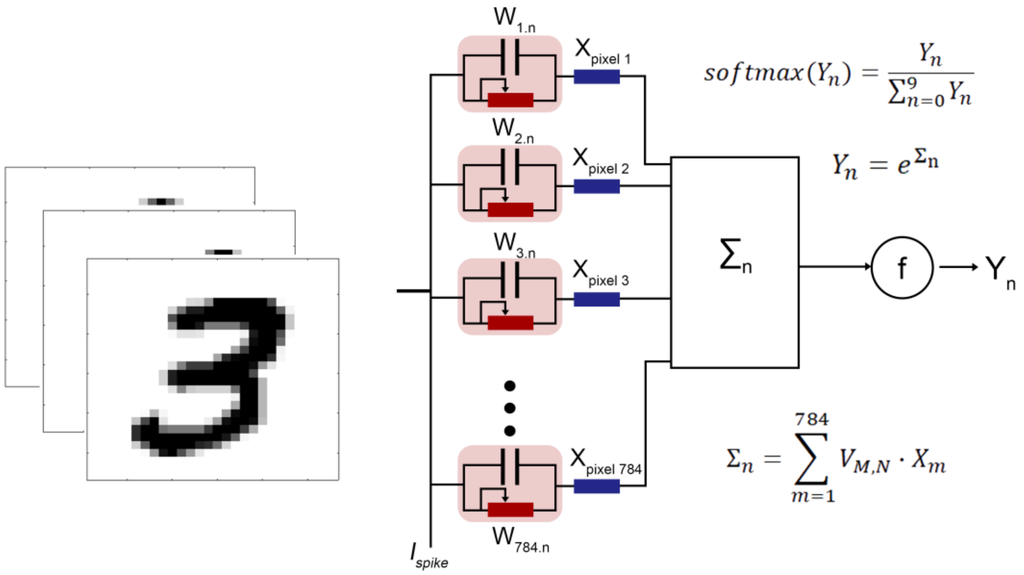

Supplementary Fig. 17 An equivalent representation of synaptic weight calculation using FMES artificial synaptic devices PSV ($W_{m,n}$).

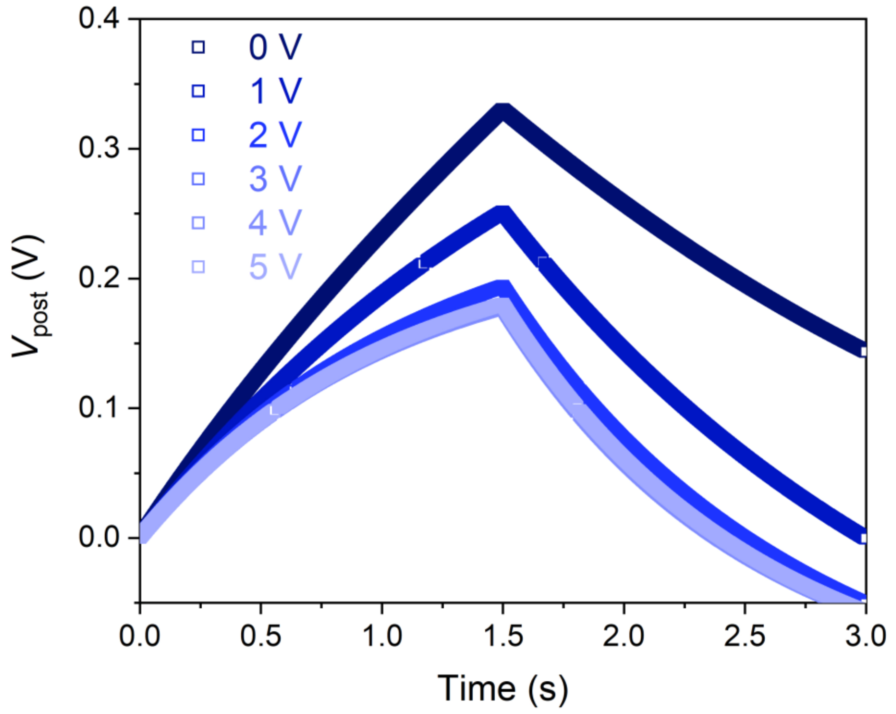

Supplementary Fig. 18 The verification results of conceptual hardware neural network.

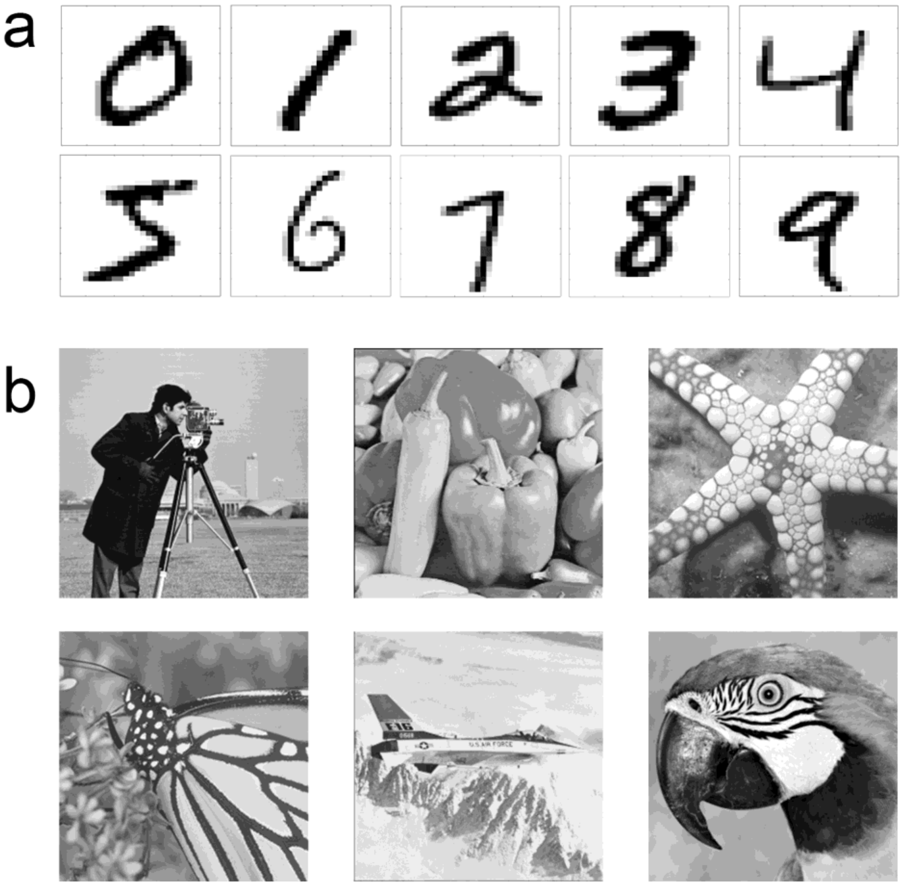

Supplementary Fig. 19 (a) The 10 type of sample in the Modified National Institute of Standards and Technology (MNIST) dataset. (b) 256×256-pixel gray images sample dataset.

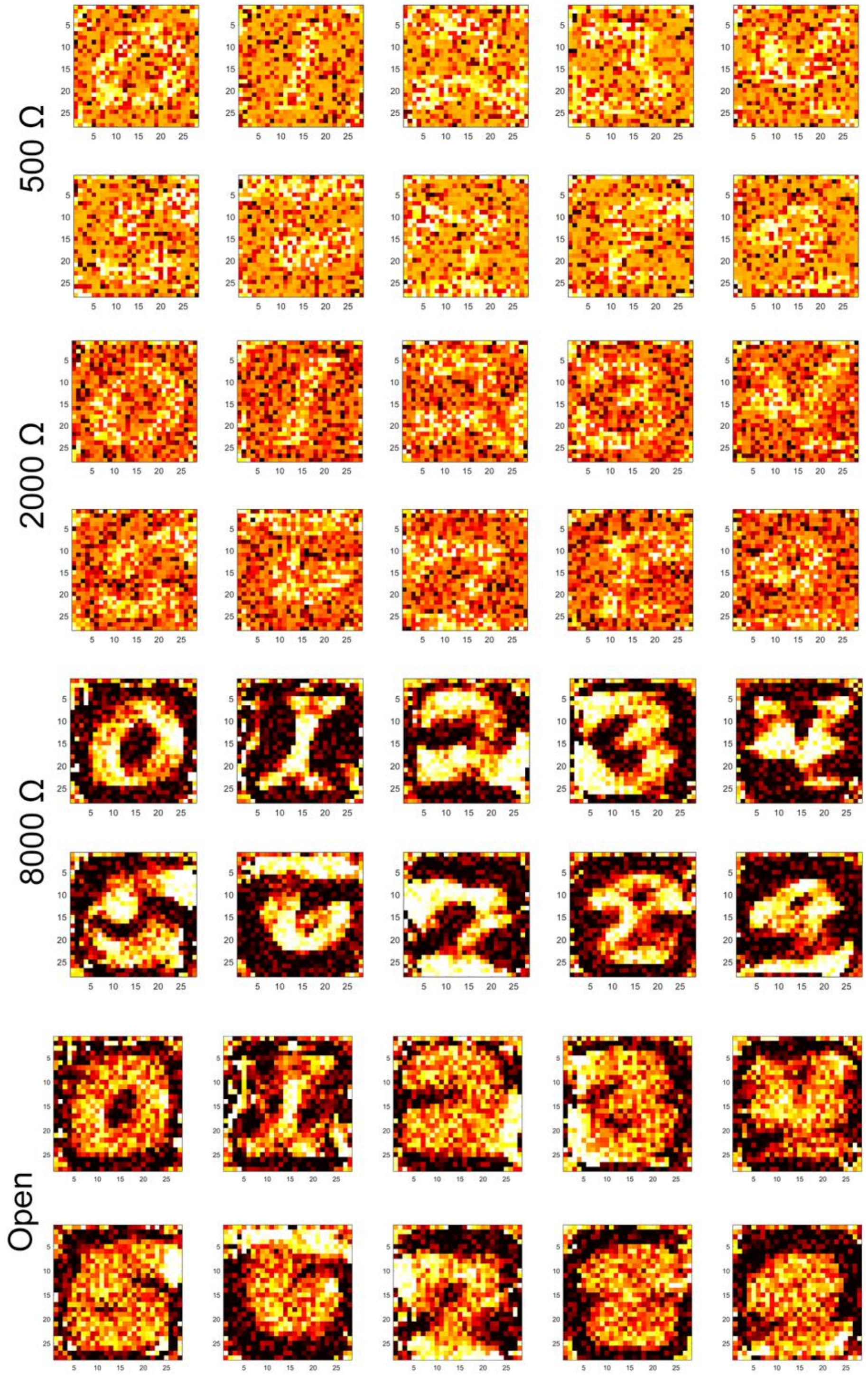

Supplementary Fig. 20 After 100 times of training phases, the weight mapping of all numbers.

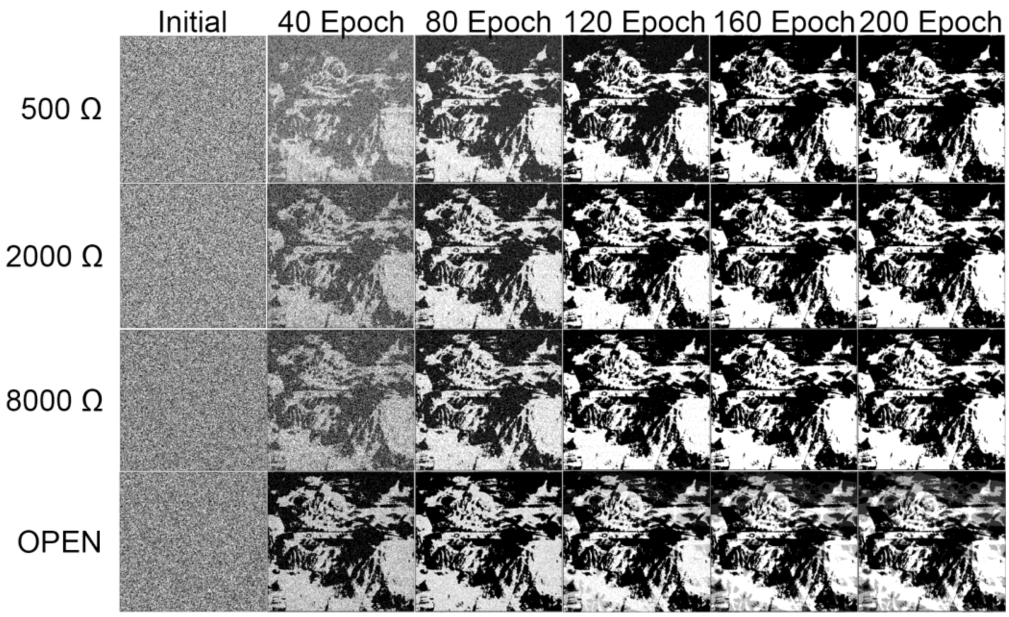

Supplementary Fig. 21 Mapping synaptic weight of another airplane image at initial, 40, 80, 120, 160 and 200 training cycle with four different LTP/LTD resistance state. In the grayscale image, white (minimum) and black (maximum) correspond to the lowest and highest synaptic weight.

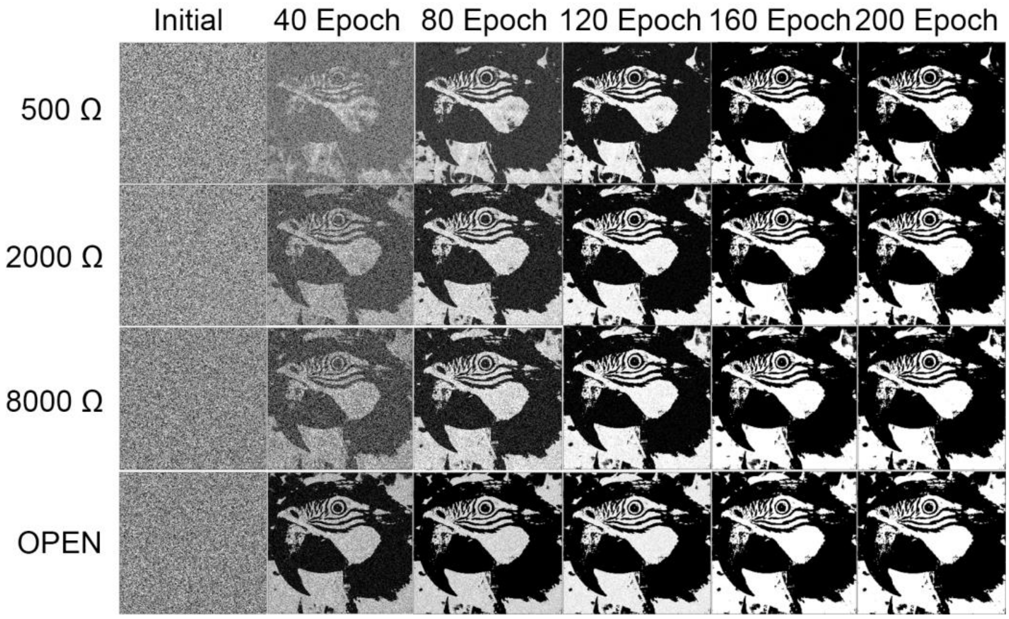

Supplementary Fig. 22 Mapping synaptic weight of another bird image at initial, 40, 80, 120, 160 and 200 training cycle with four different LTP/LTD resistance state. In the grayscale image, white (minimum) and black (maximum) correspond to the lowest and highest synaptic weight.

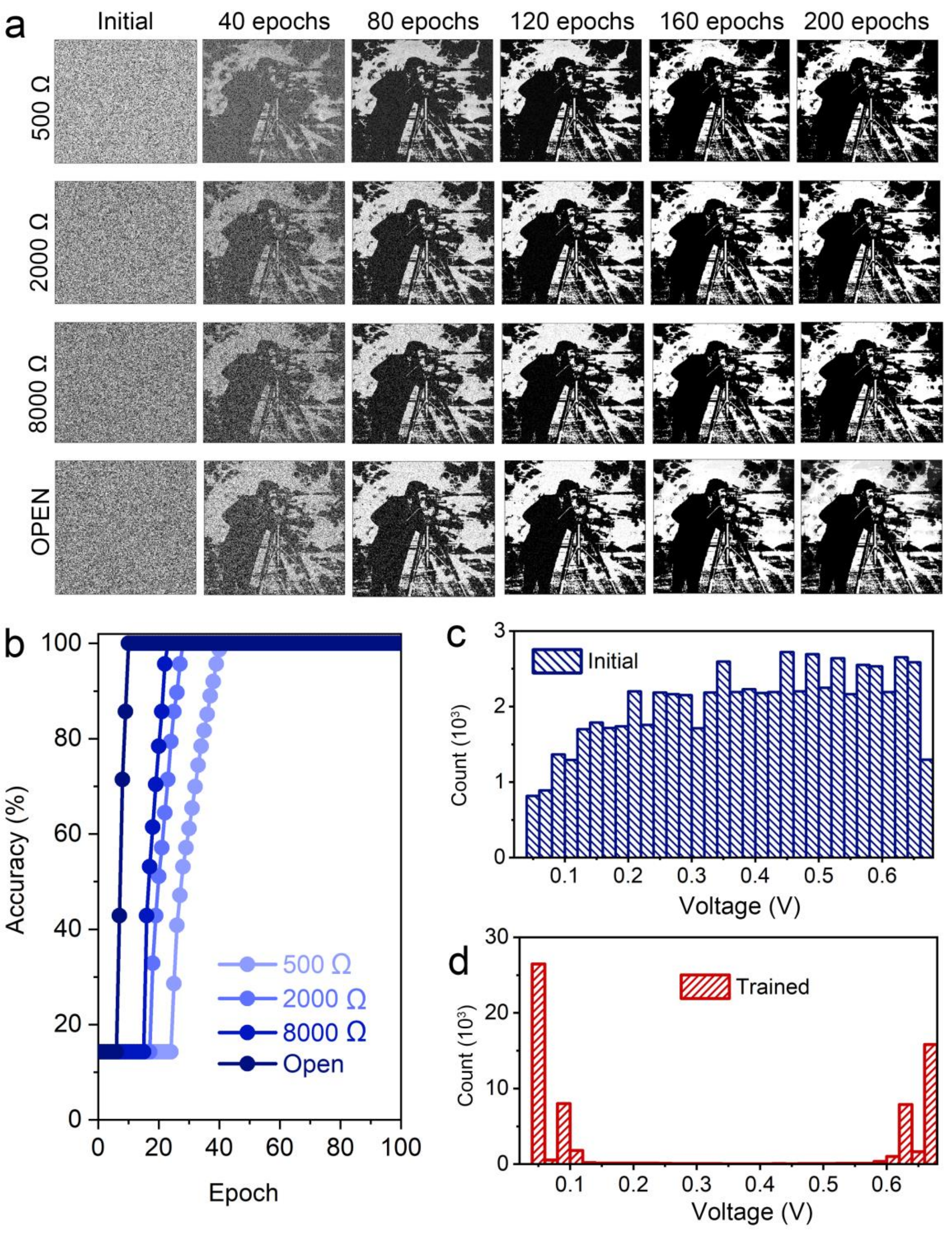

Supplementary Fig. 23 (a) Mapping synaptic weight of the input image at initial, 40, 80, 120, 160 and 200 training cycle with four different LTP/LTD resistance state. (b) Comparison of classification accuracy rates of testing image for four types of experimental dataset, with different LTP/LTD resistance states (500 Ω, 2000 Ω, 8000 Ω, and OPEN states). In the grayscale image, white (minimum) and black (maximum) correspond to the lowest and highest synaptic weight. Distribution of the FMES artificial synaptic devices PSV before (c) and after training (d).

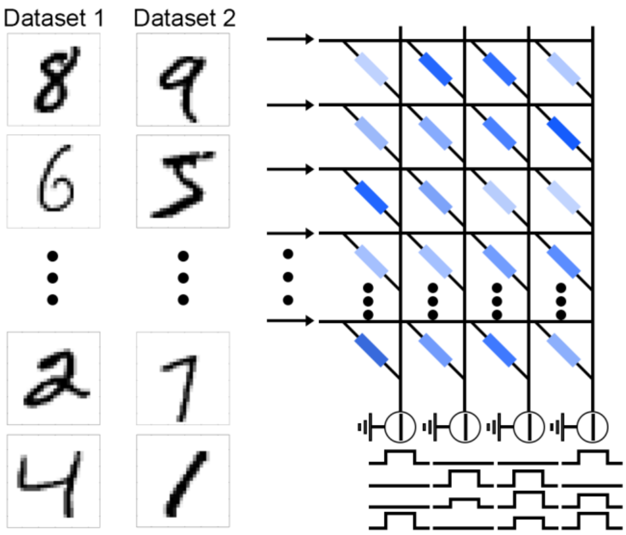

Supplementary Fig. 24 The dataset 1, including handwritten digits ‘8’, ‘6’, ‘2’, ‘4’ and dataset 2, including handwritten digits ‘9’, ‘5’, ‘7’, ‘1’ extracted from MNIST and the constructed neural network.

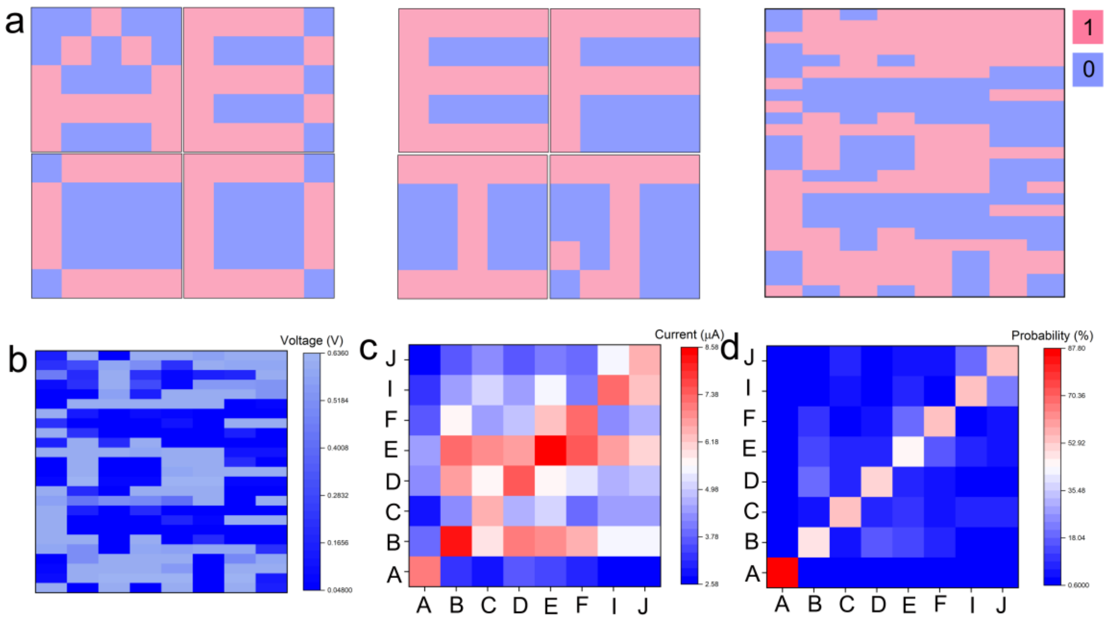

Supplementary Fig. 25 (a) The data matrix for 8 target images ‘ABCDEFIJ’; (b) Weight voltages stored in the matrix; (c) Output current after matrix operation; (d) Probability after softmax operation.
